# An siRMSD parameter of structural distortion induced by chemical modification is predictive of the off-target effect of siRNA

**DOI:** 10.1016/j.omtn.2025.102693

**Published:** 2025-09-16

**Authors:** Seongjin An, Kohei Nomura, Yoshiaki Kobayashi, Yasuaki Kimura, Hiroshi Abe, Kumiko Ui-Tei

**Affiliations:** 1 Department of Computational Biology and Medical Sciences, Graduate School of Frontier Sciences, The University of Tokyo, Chiba 277-8561, Japan; 2 Department of Chemistry, Graduate School of Science, Nagoya University, Aichi 464-8602, Japan; 3 Department of Biological Sciences, Graduate School of Science, The University of Tokyo, Tokyo 113-0033, Japan; 4 NucleoTIDE and PepTIDE Drug Discovery Center, Institute of Biomedical Engineering, Institute of Science Tokyo, Tokyo 113-8510, Japan

**Keywords:** MT: Bioinformatics, siRNA, off-target effect, chemical modifications, AGO2, computational simulation, siRMSD, structural parameter

## Abstract

We developed siRMSD, a predictive parameter for off-target effects induced by chemical modifications, to optimize siRNA therapeutics. In RNA interference, small interfering RNA (siRNA) suppresses gene function by degrading mRNA with perfect sequence complementarity, providing therapeutic potential through the targeted inhibition of disease-related genes. However, off-target effects on unintended mRNAs pose a significant challenge to clinical application. While chemical modifications improve nuclease stability and reduce off-target effects, the underlying mechanisms remain unclear. Here, we show that structural distortions caused by chemical modifications determine off-target effects. Modifications, including 2′-O-methoxyethyl, 2′-O-methyl, and 2′-formamido, at positions 2–5 disrupted the A-form RNA duplex on argonaute 2, preventing stable binding to target mRNA. In contrast, modifications at positions 6–8 had minimal impact on off-target effect resulting from changes in thermodynamic stability.

## Graphical abstract

**Figure:**
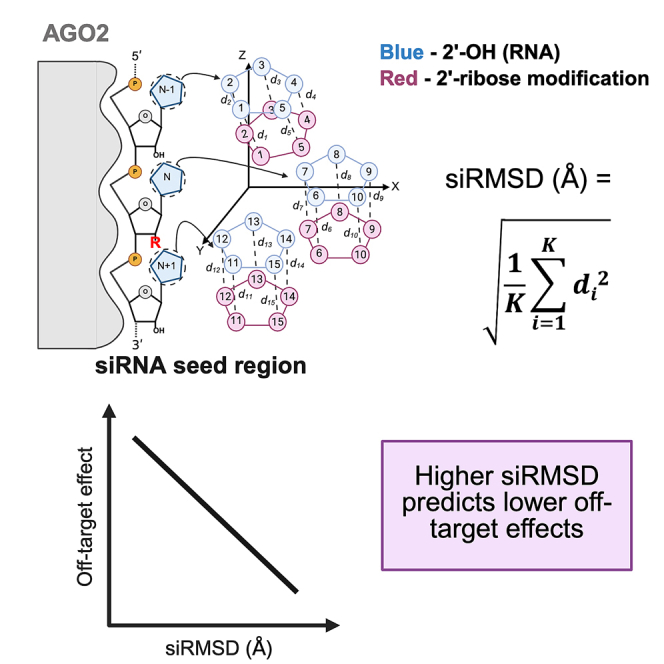

## Teaser

Chemical modifications at the seed region of siRNA induce structural distortion of the guide strand when bound to AGO2. Ui-Tei and colleagues developed siRMSD, a predictive parameter that correlates with off-target effects, to support the rational design of siRNAs.

## Introduction

RNA interference (RNAi) is a natural defense mechanism against exogenous genes triggered by small interfering RNAs (siRNAs), which are double-stranded RNAs of about 21 nucleotides with two-nucleotide 3′ overhangs.^1^^,^^2^^,^^3^ Upon entering a cell, siRNA is loaded onto argonaute 2 (AGO2) protein, forming an RNA-induced silencing complex (RISC).^4^ In the RISC, siRNA unwinds into single-stranded guide and passenger RNAs. The guide strand bound to AGO2 recognizes target mRNA with perfect complementarity, and AGO2 cleaves the mRNA, thereby silencing the target gene.^5^^,^^6^^,^^7^ RNAi has considerable therapeutic potential based on its ability to repress disease-related genes in a sequence-specific manner, including those related to rare and genetic diseases,^8^^,^^9^ and six siRNAs have been approved by the U.S. Food and Drug Administration to date.^10^^,^^11^

However, siRNA therapy has a risk of sequence-dependent off-target effects. siRNAs can suppress the expression of unintended mRNAs with partial sequence complementarities with the siRNA seed region (nucleotides 2–8 from the 5′ end of the guide RNA).^12^^,^^13^^,^^14^^,^^15^ Indeed, siRNA off-target effects can affect phenotype and changes in cell viability or toxicity are linked to particular siRNA sequence motifs.^16^^,^^17^ Most siRNAs currently on the market are fully chemically modified, with all 2′ positions substituted with either 2′-deoxy-2′-fluoro (2′-fluoro) or 2′-O-methyl (2′-OMe), and with terminal phosphorothioates added for stability.^18^^,^^19^ However, these chemical modifications can also be employed to mitigate off-target effects, primarily through two distinct mechanisms.

The first approach is to introduce chemical modifications that disrupt the guide RNA structure on the AGO2 protein, preventing stable binding between siRNA and off-target mRNAs. Therefore, it is important to clarify the effects of chemical modifications on RNAi or off-target activities for siRNA drug development. Previous studies have shown that siRNAs modified with 2′-OMe in the seed region result in the reduction of off-target effects.^20^^,^^21^ Structural simulation of modification at the third position from the 5′ end of the guide RNA with 2′-OMe suggested that the structural distortion of the nucleotide at the fourth position may cause the reduced off-target effects.^20^ Other modification, 2′-O-methoxyethyl (2′-MOE), at position 2 is also shown to reduce off-target effects.^22^ These studies may indicate that steric size modifications larger than 2′-hydroxyl (2′-OH) in the seed position can reduce off-target effects.

The second is to decrease thermodynamic stability, thereby reducing the binding affinity between the siRNA seed region and the off-target mRNA in the RISC. The off-target effect of an unmodified siRNA is shown to be positively correlated with the thermodynamic stability of the protein-free RNA duplex formed between the seed region and the off-target mRNA.^23^ Although chemical modifications usually enhance the stability, specificity, or immunogenicity of siRNAs,^24^^,^^25^^,^^26^^,^^27^^,^^28^^,^^29^^,^^30^ several chemical modifications can reduce the *T*_*m*_ value.^9^ The 2′-deoxy (DNA) modification is less stable than unmodified RNA. In fact, sequential modifications of DNA in the seed region moderately reduce off-target effects.^20^^,^^31^

Several *in silico* studies have examined how chemical modifications influence the structure of RNA,^32^ and, during the preparation of this manuscript, it was reported how these structural changes related to RNAi activity.^33^ However, most of these analyses have been limited to protein-free duplex structures. While these models offer insight into the structural features of modified siRNAs before loading on AGO2 protein, they do not capture the structural context on AGO2 within the RISC, where off-target effects are regulated.

We recently reported a new chemical modification, 2′-formamido (2′-FA), which significantly decreases thermodynamic stability.^34^ Introduction of 2′-FA modifications at any position and on any type of nucleotide within the siRNA seed region reduced off-target effects. The magnitude of the reduction of off-target effects varied depending on the positions in the seed region. However, it remains unclear whether these differences are attributable to the structural distortion or changes in the thermodynamic stability induced by chemical modifications.

**Figure 1:**
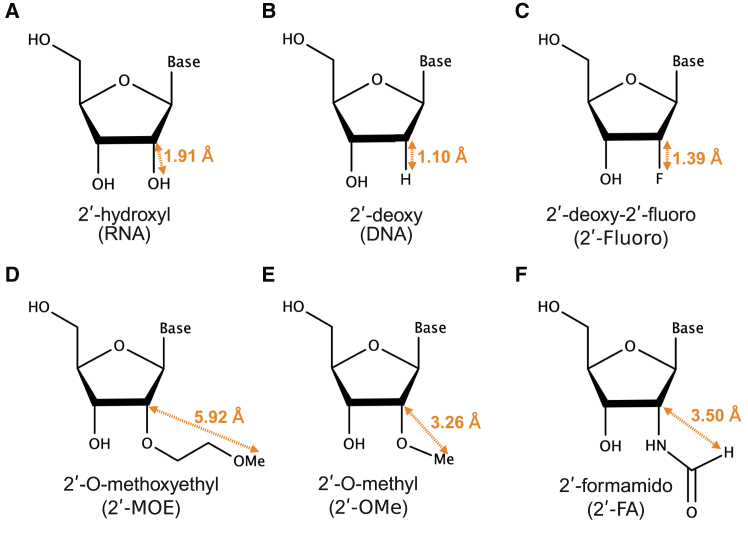
Structures of unmodified RNA and 2′-ribose modifications used in this study (A) 2′-hydroxyl (RNA), (B) 2′-deoxy (DNA), (C) 2′-deoxy-2′-fluoro (2′-fluoro), (D) 2′-O-methoxyethyl (2′-MOE), (E) 2′-O-methyl (2′-OMe), and (F) 2′-formamido (2′-FA). The distances between the C2 carbon atom of the ribose furanose ring and the farthest atom in the 2′-ribose modifications are shown by measured from the computer-simulated structures (Figures S5–S11), and depicted for indicating the modification sizes.

To better understand these positional effects and to examine the structural changes induced by chemical modifications on the AGO2 protein, we evaluated the properties of 2′-FA compared to several other chemical modifications—DNA, 2′-fluoro, 2′-MOE, and 2′-OMe—which have varying steric sizes and thermodynamic stabilities (Figure 1).^9^^,^^35^^,^^36^ To determine the mechanism behind the reduction of off-target effects induced by steric hindrance, we investigated the structural changes through computational simulation. As a result, we clarified the relationship between the structures of siRNAs on AGO2 proteins and the extent of reduction in off-target activity. In this study, we then propose a new parameter, siRMSD, to estimate the reduction of siRNA off-target effects due to chemical modifications.

## Results

### RNAi and off-target activities of siRNAs with chemical modifications

**Figure 2:**
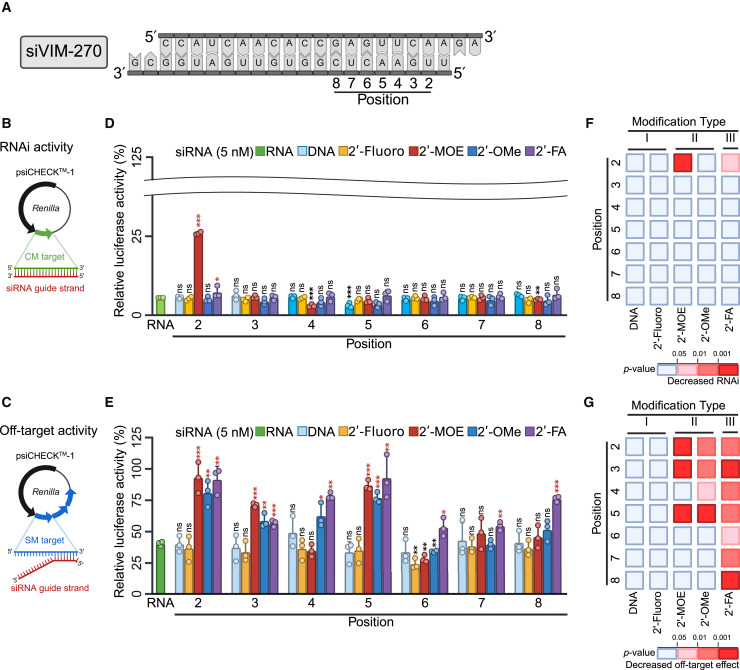
Schematic of reporter constructs for RNAi and off-target activity assays of chemically modified siRNAs (A) Sequence of siVIM-270 and the positions. The upper RNA strand represents the passenger strand, and the lower represents the guide strand. (B and C) The reporter construct for RNAi activity assay was psiCHECK-1 with one CM target sequence of siRNA in the 3′-UTR of *Renilla luciferase* (B), and that for off-target activity was psiCHECK-1 with three tandem repeats of the SM sequence (C). (D and E) Results of reporter assays of (D) RNAi and (E) off-target activities at 5 nM siRNAs. Dose dependent effects of siVIM-270 were shown in Figure S1. Green indicated unmodified siRNA; light blue, DNA; yellow, 2′-fluoro; red, 2′-MOE; dark blue, 2′-OMe; purple, 2′-FA. Vertical bar indicated relative luciferase activity, normalized to siCont. Horizontal bar, position of a chemical modification. The experiments were conducted in triplicate. Eorror bars represent standard deviation (SD) of three independent experiments. The modified siRNAs were compared to their unmodified counterparts using the *t* test. Red asterisk, significant decrease in activity compared to the unmodified counterpart; black asterisk, significant increase in activity. ∗*p* < 0.05, ∗∗*p* < 0.01, ∗∗∗*p* < 0.001. Heatmaps showed the effects on RNAi (F) and off-target (G) activities. Modification type I, DNA, and 2′-fluoro; type II, 2′-MOE and 2′-OMe; type III, 2′-FA.

To investigate the effects of chemical modifications of siRNAs on RNAi and off-target activities, we used five chemical modifications—DNA, 2′-fluoro, 2′-MOE, 2′-OMe, and 2′-FA (Figure 1).^34^ Each modification was introduced into the seed region of siVIM-270 targeting *vimentin* gene (Figure 2A). To evaluate the effect of each chemical modification on RNAi and off-target activities, we conducted luciferase reporter assays. RNAi activity was measured using a reporter construct with the completely-matched (CM) target sequence of an siRNA in the 3′-UTR of the *Renilla luciferase* gene (Figure 2B), and off-target activity was measured using a reporter construct with three tandem repeats of the seed-matched (SM) sequence in the 3′-UTR of *Renilla luciferase* (Figure 2C). Each siRNA at 0.005, 0.05, 0.5, or 5 nM was transfected with the firefly and *Renilla* luciferase expression constructs, and *Renilla* luciferase activity was normalized to that of firefly luciferase to calculate RNAi or off-target activity (Figure S1).

Because the magnitudes of RNAi and off-target effects increased with increasing siRNA concentration (Figure S1), we compared the results of the highest concentration (5 nM) in Figures 2D and 2E siRNAs modified with DNA, 2′-fluoro, and 2′-OMe at any position in the guide strand had little effect on RNAi activity and had comparable activities to unmodified siRNA (Figure 2D). In contrast, the introduction of 2′-MOE at position 2 significantly reduced RNAi activity relative to unmodified siRNA, while 2′-FA exhibited a slight effect. These results suggested that RNAi activity is related to the size of the 2′-ribose modification that was defined as the length between the C2 carbon atom of the ribose furanose ring and the furthest atom in the modifying group (Figure 1). The largest modifications, 2′-MOE (5.92 Å) and 2′-FA (3.50 Å), reduced RNAi activities, but smaller modifications—DNA (1.10 Å), 2′-fluoro (1.39 Å), and 2′-OMe (3.26 Å)—did not (Figures 1, 2D, and 2F).

Regarding off-target activity, DNA, and 2′-fluoro modifications had little effects compared to unmodified siRNAs (Figure 2E). The 2′-MOE modification reduced off-target activities at positions 2, 3, and 5, and 2′-OMe modification reduced off-target activities at positions 2–5. siRNAs modified with 2′-FA throughout the seed region showed reduced off-target activities. Therefore, chemical modifications can be classified according to their off-target activities (Figures 2E and 2G). Modification type I, including DNA and 2′-fluoro, has negligible effects on off-target activity. Modification type II includes 2′-MOE and 2′-OMe, which reduced off-target activities at positions 2, 3, and 5, and 2–5, respectively, with minimal effects at positions 6–8. In contrast, modification type III, represented by 2′-FA, reduces off-target effects at any position within the seed region.

### Validation of RNAi and the off-target effects of siRNAs with chemical modifications by quantitative reverse-transcriptase polymerase chain reaction and microarray analyses

To validate the effects of 2′-ribose modifications (DNA, 2′-fluoro, 2′-MOE, 2′-OMe, and 2′-FA) on an endogenous target and off-target transcripts, quantitative reverse-transcription polymerase chain reaction (RT-qPCR) and microarray analyses were conducted. For this purpose, we used siRNAs with chemical modifications at position 2 (siVIM-270_DNA_2, _fluoro_2, _MOE_2, _OMe_2, and _FA_2) and unmodified siVIM-270, because modifications at position 2 of the guide strands had marked effects on off-target activities (Figure 2E). Each siRNA was transfected into HeLa cells at 50 nM, and total RNA was purified from the cells 1 day later.

**Figure 3:**
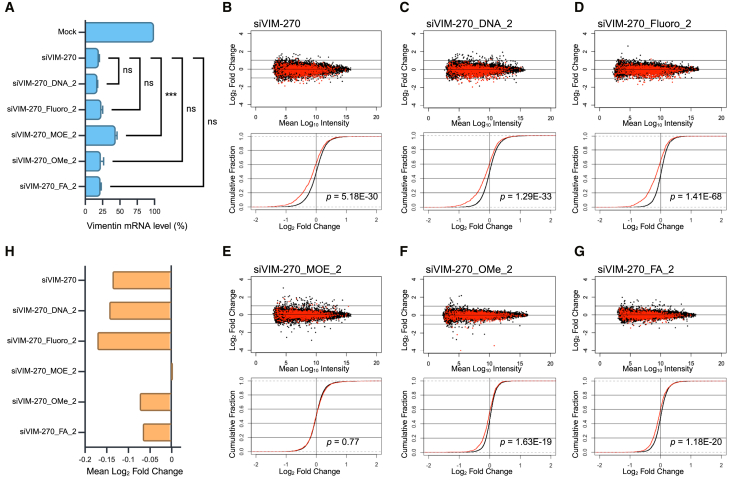
Expression levels of *vimentin* mRNA and off-target mRNAs after transfection of siVIM-270 with chemical modifications at position 2 (A) mRNA levels of *vimentin* after transfection of siVIM-270, siVIM-270_DNA_2, siVIM-270_fluoro_2, siVIM-270_MOE_2, siVIM-270_OMe_2, and siVIM-270_FA_2 compared to mock, as determined by qRT-PCR. Error bars represent standard deviation (SD) of three independent experiments. One-way ANOVA relative to unmodified siVIM-270 was performed. ∗*p* < 0.05, ∗∗*p* < 0.01, ∗∗∗*p* < 0.001. (B–G) MA plots (top) and the corresponding cumulative distribution plots (bottom) of microarray results. In the MA plots, the vertical axis shows the log_2_ fold change of the signal intensity of each transcript in the cells transfected with siVIM-270 or its modified siRNA compared to that in the mock-transfected cells; horizontal axis, average log_10_ signal intensity of each transcript in cells transfected with siVIM-270 and its modified siRNAs and that in the mock-transfected cells. Red dots, 1185 off-target transcripts with the siVIM-270 SM sequence in their 3′-UTRs; black dots, 11,919 non-off-target transcripts without the SM sequence. In the cumulative distributions, the horizontal axis represents the log_2_ fold change and the vertical axis shows the cumulative fraction. Red line: off-target transcripts; black line: non-off-target transcripts. *p* values were calculated by Wilcoxon rank-sum test. (H) Seed-dependent off-target effects measured by microarrays, shown as mean log_2_ fold changes of transcripts in the cells transfected with siVIM-270 with chemical modifications compared to those with unmodified siVIM-270.

The expression levels of the target gene of these siRNA, *vimentin*, were measured by RT-qPCR. All of the siRNAs, except for siVIM-270_MOE_2, suppressed the expression of the target gene at the similar levels to unmodified siVIM-270 (Figure 3A), partially consistent with the results of reporter assays (Figure 2D). Next, the effects on off-target genes were validated by microarray and RT-qPCR analyses. MA plots of the microarray data showed the log_2_ changes in expression levels in cells transfected with the siRNAs compared to mock transfected cells (Figures 3B–3G). The cumulative distribution represents the cumulative fraction curves of the average fold changes of the transcripts with and without SM sequences in their 3′-UTRs. The differences in the mean log_2_ fold changes between SM and non-SM transcripts of the siVIM-270 guide strand are indicative of the magnitudes of off-target effects (Figure 3H). siVIM-270_DNA_2 and siVIM-270_Fluoro_2 had off-target effects at similar magnitude with unmodified siVIM-270, whereas siVIM-270_MOE_2 had a negligible off-target effect, and siVIM-270_OMe_2 and siVIM-270_FA_2 had reduced but weak off-target effects (Figure 3H), in agreement with the results of reporter assays (Figure 2E). In microarray analyses using siRNAs with 2′-MOE and 2′-FA at position 4 (siVIM-270_MOE_4 and _FA_4), siVIM-270_MOE_4 showed strong off-target effects but siVIM-270_FA_4 weak effects (Figure S2), in consistent with reporter assays (Figure 2E). The microarray data closely aligned with the RT-qPCR results for endogenous off-target genes (*vesicle-associated membrane protein-associated protein A* [*VAPA*], *myotrophin* [*MTPN*], *protein tyrosine phosphatase receptor type F* [*PTPRF*]), and a non-target gene (*surfeit 4* [*SURF4*]) (Figure S3A). Notably, the RT-qPCR and microarray data were strongly correlated (*r* = 0.93) (Figure S3B). These results indicated that the effects of 2′-ribose modifications on RNAi and off-target effects against for endogenous genes were essentially consistent with results observed by reporter assays.

### Computational simulations of siRNA structures with chemical modifications at positions 2–8

The magnitudes of off-target activities depended differently on the positions and types of modifications (Figures 2E, S1, 3H, and S2F). The introduction of chemical modifications at a particular site can induce structural changes in siRNAs on the AGO2 protein.^20^^,^^37^^,^^38^ To evaluate the correlation between structure and off-target effect, we conducted computational simulations to investigate the structural changes caused by the chemical modifications. The structures of siRNA guide strands on AGO2, as registered in the Protein DataBank (PDB) IDs 4F3T and 4W5O, were used as original structures,^39^^,^^40^ and simulation was performed using density functional theory (DFT) at the ωB97X-D/6–31G(d) level. Each modification was introduced into each RNA at positions 2–8 of the seed region, and the modified RNA and its nearest neighbor RNAs (one upstream and downstream nucleotides) were included in the simulations (Figures S4A–S4F), because the introduction of a 2′-OMe modification at position 3 results in structural changes at the adjacent nucleotide.^20^ RNAs at nucleotide positions 7–10 from the 5′ end of the siRNA guide strand were included in structural simulations of position 8 (Figure S4G). In addition, amino acids of AGO2 predicted to interact with guide RNAs <4 Å in either PDB ID 4F3T or 4W5O were included for simulation. To simulate the structural changes caused by modifications at position 5, PDB ID 4F3T was used, whereas PDB ID 4W5O was used for other positions. The PDB IDs of the original structures and the amino acids included in the simulations are listed in Table S1. Previous studies have shown that the overall conformation of AGO2 remains largely unchanged when fully modified siRNA is introduced.^37^ Based on this observation, we fixed the central carbon (Cα) atom of each amino acid to minimize structural changes in the AGO2 protein when optimizing the geometry of the structures.

The results of simulations of siRNA structures on AGO2 are shown in Figures S5–S11. In the obtained structures, the notable points affected by 2′-ribose modification at each of positions 2–8 are highlighted with red dashed squares and enlarged in Figure 4.

**Figure 4:**
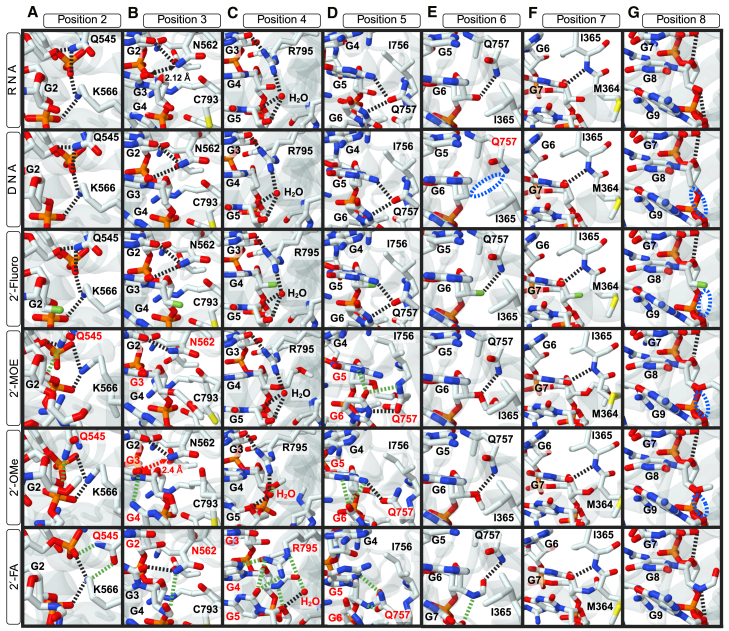
Results of computational simulations of unmodified nucleotides or nucleotides with chemical modifications at positions 2–8 in siRNA seed region These figures show enlargements of red squares from Figures S5–S11, highlighting nucleotide positions (A–G) (positions 2–8 from the 5′ end of the siRNA guide strand) that were affected by the 2′-ribose modifications. From upper to bottom, unmodified RNA, DNA, 2′-fluoro, 2′-MOE, 2′-OMe, or 2′-FA modification. Dashed line, hydrogen bond. The structures were optimized by fixing the alpha carbons of the amino acids listed in Table S1. Green dashed lines, changed hydrogen bond patterns. The names of changed amino acids, nucleobases, and water molecules involved in hydrogen bond were highlighted in red. Blue dashed ellipse, missing hydrogen bonds.

In the simulated structures of chemical modifications at position 2, the phosphate group of the third nucleotide formed an electrostatic interaction with lysine 566 (K566) of AGO2 (Figure 4A). The molecular diameters of 2′-MOE, 2′-OMe, and 2′-FA were greater than 2′-OH in the unmodified RNA (1.91 Å) (Figure 1). When these modifications were introduced at position 2, a steric clash with K566 was induced, leading to a positional shift of the nucleotide to alter the hydrogen bonding pattern of glutamine 545 (Q545). Conversely, the DNA and 2′-fluoro modifications did not disrupt the hydrogen bonding pattern of Q545, and their structures were similar to the unmodified RNA.

Since siRNAs undergo phosphorylation before being loaded onto AGO2,^41^ we examined whether this suppression of RNAi activity was due to impaired phosphorylation. To test this, we compared four types of chemically synthesized siRNAs: (1) unmodified siRNA without a 5′-phosphate, (2) unmodified siRNA with a 5′-phosphate, (3) siRNA with a 2′-MOE modification at position 2 but without a 5′-phosphate, and (3) siRNA with both a 2′-MOE modification at position 2 and a 5′-phosphate (Figure S12). The results showed that while 5′-phosphorylation significantly enhanced RNAi activity, siRNAs with a 2′-MOE modification at position 2 exhibited reduced RNAi efficiency regardless of phosphorylation. These findings suggest that the inhibition of RNAi activity is not due to impaired phosphorylation but rather steric hindrance caused by the bulky 2′-MOE modification.

In the simulated structures of chemical modifications at position 3, modifications of large steric sizes, such as 2′-MOE or 2′-FA, caused a steric clash with cysteine 793 (C793), resulting in a positional shift of nucleotide 3 (Figure 4B). This altered the hydrogen bonding pattern of asparagine 562 (N562) with the nucleobases of nucleotides 2 and 3, leading to structural changes. Although 2′-OMe did not alter this hydrogen bonding pattern, it increased the distance of hydrogen bonds between N562 and nucleobase 3 from 2.12 Å to 2.4 Å. The result was significant structural changes at the position of nucleobase 4, consistent with the previous report.^20^ DNA and 2′-fluoro did not show such changes in the hydrogen bonding pattern or nucleotide positions.

In the simulated structures of modifications at position 4, only siRNAs with 2′-OMe or 2′-FA caused structural changes (Figure 4C). In this simulation, arginine 795 (R795) interacts with the nucleobase of nucleotide 3 and a water molecule close to the ribose sugar of nucleotide 5. However, the introduction of 2′-OMe or 2′-FA into nucleotide 4 shifted the position of the water molecule, altering its hydrogen bonding pattern with the ribose sugar of nucleotide 5. In addition, the hydrogen bonding pattern of R795 was altered by 2′-FA. The changes in the positions of the water molecule and hydrogen bonds likely caused structural changes compared to the unmodified RNA. Because DNA, 2′-fluoro, and 2′-MOE at this position cannot alter the position of the water molecule, no structural change occurred.

Despite the large steric size of 2′-MOE (Figure 1), it did not cause steric hindrance at position 4. Notably, histidine 753 (H753), alanine 754 (A754), glycine 755 (G755), R795, serine 796 (S796), valine 797 (V797), and serine 798 (S798) of AGO2 were estimated to form a cavity-like structure near the 2′-OH of nucleotide 4 (Figure S13A). The computational simulation revealed that H753, R795, and S798 form a similar cavity-like structure, and the 2′-MOE of nucleotide 4 stably resides within this cavity (Figures S13B and S13C). Consequently, 2′-MOE modification does not cause significant structural changes. By contrast, 2′-OMe and 2′-FA do not reach this cavity and therefore interact with different amino acids to induce steric hindrance.

At position 5, glutamine 757 (Q757) formed hydrogen bonds with the nucleobases of nucleotides 5 and 6 (Figure 4D). Isoleucine 756 (I756) was positioned close to the 2′-ribose modification of nucleotide 5. The steric modifications 2′-MOE, 2′-OMe, and 2′-FA (Figure 1), created steric clashes with I756, causing positional shifts of nucleotide 5 and altering the hydrogen bonding pattern of Q757. These changes in hydrogen bonding led to structural changes in the siRNA and reduced off-target effects.

The structural changes caused by the 2′-ribose modifications occurred primarily at positions 2–5. The modifications at positions 6–8 did not cause significant structural changes compared to the unmodified RNAs (Figures 4E–4G and S9–S11). At position 6, the absence of a 2′-hydroxyl group in DNA resulted in the loss of a hydrogen bond with Q757 (Figure 4E, second from the top), and 2′-FA at nucleotide 6 formed a hydrogen bond with the oxygen of the furanose ring at nucleotide 7 (Figure 4E, bottom). However, these changes did not affect the nucleotide structures. At position 7, chemical modifications did not cause structural changes (Figure 4F). A kink between nucleotides 6 and 7 of the guide RNA is observed in the target-unbound state. Upon target binding, this kink is resolved by a shift in the helix-7 domain of AGO2, particularly involving methionine 364 (M364) and isoleucine 365 (I365), to allow base pairing with the target RNA.^39^^,^^42^^,^^43^ Therefore, to simulate modifications of nucleotides 6 and 7, the Cα atoms of M364 and I365 were excluded from being fixed. Previous studies have shown that certain sugar modifications, such as α-(L)-threofuranosyl nucleic acid (TNA), at position 7 can influence RNA structure. TNA has been reported to induce structural distortions at this position, which were attributed to its unique 3′-2′ linkage and sugar connectivity rather than a conventional ribose backbone.^44^ In contrast, the 2′-ribose modifications used in this study do not alter the sugar-phosphate backbone connectivity and, as a result, did not induce comparable structural changes. At position 8, 2′-fluoro, DNA, 2′-MOE, and 2′-OMe did not form hydrogen bonds with the oxygen atoms of the furanose rings of nucleotide 9 (Figure 4G). Nonetheless, superimposition of the structures of the unmodified and modified RNA indicated negligible structural changes (Figure S14). Therefore, the effects of chemical modifications on the structural changes at positions 6–8 differed from those at positions 2–5.

The results of computational simulations suggested that modification type I (small-sized modification) caused negligible structural distortions in the seed region. However, modification types II and III (large-sized modifications) caused structural distortions at positions 2–5.

### Superimposed structures of siRNA guide strands on AGO2s in PDB

To investigate the structural changes occurring specifically at positions 2–5 in the siRNA guide strands, we compared siRNA structures on AGO2 registered in PDB. In addition to the structures used for simulations (PDB IDs: 4W5O^39^ and 4F3T^40^), the structures of other AGO2-guide RNA complexes (PDB IDs: 4OLA^43^ and 8D71^45^) were superimposed (Figure S15); they were aligned by fixing the Cα atoms of the Q545, N562, K566, I756, Q757, C793, and R795, which were predicted to affect structural changes by chemical modifications (Figures 4A–4D). Although they have different guide RNA sequences, the nucleotides at positions 2–5 of the guide RNAs occupied nearly identical positions in the structures (Figure S15A), while the nucleotides at positions 6–8 exhibited different alignments (Figure S15B). On the AGO2 protein, nucleotides 2–5 of the guide RNA maintained a near A-form structure, similar to double-stranded RNA, even without binding to the target mRNA. This pre-organized A-form structure reduced the entropic cost, facilitating target interaction.^46^^,^^47^ To maintain this A-form structure, nucleotides 2–5 must be fixed with several amino acid residues,^40^^,^^42^ probably including Q545, N562, K566, C793, and R795 as predicted by this study. Moreover, the guide RNA-AGO2 interaction primarily involves an interaction between the phosphate backbone or ribose sugar of the guide RNA and amino acids of AGO2,^43^ which means that the effects of 2′-ribose modifications are not sequence dependent. However, the structures of nucleotides 6–8 differ depending on their sequences (Figure S15B), because this region is not necessary to be fixed.

### siRMSD value determines the relationship between structural distortion and off-target activity

**Figure 5:**
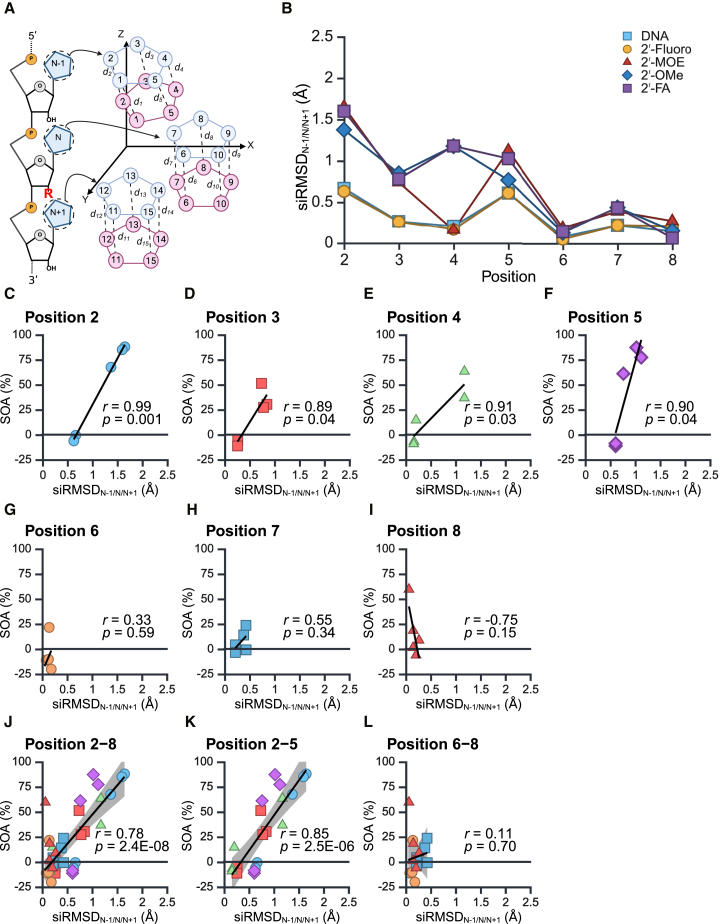
Calculation of siRMSD_N-1/N/N+1_ values and their correlations with SOAs (A) The siRMSD_N-1/N/N+1_ values were calculated using the siRMSD values at the N−1/N/N+1 positions relative to the modified nucleotide at position N, where the 2′-ribose modification was introduced at the location shown as a red uppercase “R”. (B) The siRMSD_N-1/N/N+1_ values at each position were determined for the following chemical modifications: DNA (light blue), 2′-fluoro (yellow), 2′-MOE (orange), 2′-OMe (blue), and 2′-FA (purple). (C–I) The correlation between siRMSD_N-1/N/N+1_ and SOA was shown by dot plots at each position from 2 to 8. (J–L) The correlations for positions 2–8, 2–5, and 6–8 were shown respectively. The gray-shaded areas in represent the 95% confidence intervals.

The results of computational simulations of siRNA structures (Figure 4) and off-target reporter/microarray assays (Figures 2E, 3H, and S2F) suggested that the pre-organized near A-form configuration of the guide RNA on AGO2 facilitates target binding, but 2′-ribose modifications might cause structural distortions and hampering target binding. Based on this hypothesis, we quantified the structural changes by calculating the root-mean-square deviations (RMSDs), and their correlations with off-target effects were analyzed. Usually, the RMSD value is used for quantifying the configuration of amino acids,^48^ we therefore named this quantification value for siRNA as siRMSD. The siRMSD value is the average squared distances of all atoms in the nucleobase, except for hydrogen atoms, in the modified siRNA compared to the unmodified siRNA were determined. A high siRMSD value means that the three-dimensional location of nucleobase(s) of the modified siRNA significantly moved away from its original position(s) in the unmodified siRNA. In this analysis, the nucleotide with a chemical modification was defined as position N, with the nucleotide located one nucleotide upstream defined as N−1 and that located one nucleotide downstream as N+1 (Figure 5A and S16A–S21A). To determine how structural changes of nucleotide at N−1, N, or N+1 caused by the chemical modification at nucleotide N affect off-target effects, siRMSD values were calculated across nucleotides 2–8 using all possible combinations of the N−1, N, and N+1 nucleobases: N−1 (Figure S16B), N (Figure S17B), N+1 (Figure S18B), N−1/N (Figure S19B), N−1/N+1 (Figure S20B), N/N+1 (Figure S21B), and N−1/N/N+1 (Figure 5B). On the other hand, substantial off-target activities (SOAs), which refer to the changes in off-target activity caused by chemical modifications of siRNAs compared to unmodified siRNAs, were calculated using the following Equation 1 based on the values from Figure 2E.

(Equation 1) $$\text{Substantial}\;\text{off}\;\text{target}\;\text{activity}\;\left(\text{SOA}\right)\left(\%\right)=\frac{{\text{Relative}\;\text{luc}\;\text{activity}}_{\text{modified}}-{\text{Relative}\;\text{luc}\;\text{activity}}_{\text{unmodified}}}{100-{\text{Relative}\;\text{luc}\;\text{activity}}_{\text{unmodified}}}\times 100$$

The correlations between SOAs and total siRMSD values at positions 2–8 were then analyzed (Figures 5J and S16J–S21J). The siRMSD values calculated at each position of single nucleotide (siRMSD_N-1_, siRMSD_N_, siRMSD_N+1_) showed the correlation coefficients with the SOAs at *r* = 0.72, 0.65, and 0.70, respectively (Figures S16J–S18J). The correlation coefficients for sum of the two of the three nucleotides, siRMSD_N-1/N_, siRMSD_N-1/N+1_, siRMSD_N/N+1_ were *r* = 0.72, 0.77, and 0.66, respectively (Figures S19J–S21J). Summarizing siRMSD values of all three nucleotides, the correlation coefficient of siRMSD_N-1/N/N+1_ was the highest at *r* = 0.78 (Figure 5J). Therefore, these findings indicated that the siRMSD_N-1/N/N+1_ value are the most suitable parameter for capturing structural deviations related to the reduction of off-target effects.

The correlation between the siRMSD_N-1/N/N+1_ value at each position from 2 to 8 and the corresponding SOA was calculated (Figures 5C–5I). Each nucleotide at 2–5 exhibited high correlation coefficients (0.99, 0.89, 0.91, and 0.90 for position 2, 3, 4, 5, respectively) and low *p* values (<0.05) (Figures 5C–5F). By contrast, positions 6–8 showed no significant correlations (Figures 5G–5I). Then, the correlation coefficients between siRMSD_N-1/N/N+1_ and SOAs at positions 2–8 were calculated by dividing two positions 2–5 and 6–8 (Figures 5J–5L). The correlation coefficient of siRMSD_N-1/N/N+1_ at positions 2–5 was higher (*r* = 0.85) than that at positions 2–8 (*r* = 0.78), and the positions 6–8 (*r* = 0.11) showed negligible correlation. These results indicated a strong association between siRMSD_N-1/N/N+1_ values and SOAs at positions 2–5 but not at positions 6–8.

### Validation of siRMSD as a parameter for predicting off-target activity using siRNAs with different sequences

**Figure 6:**
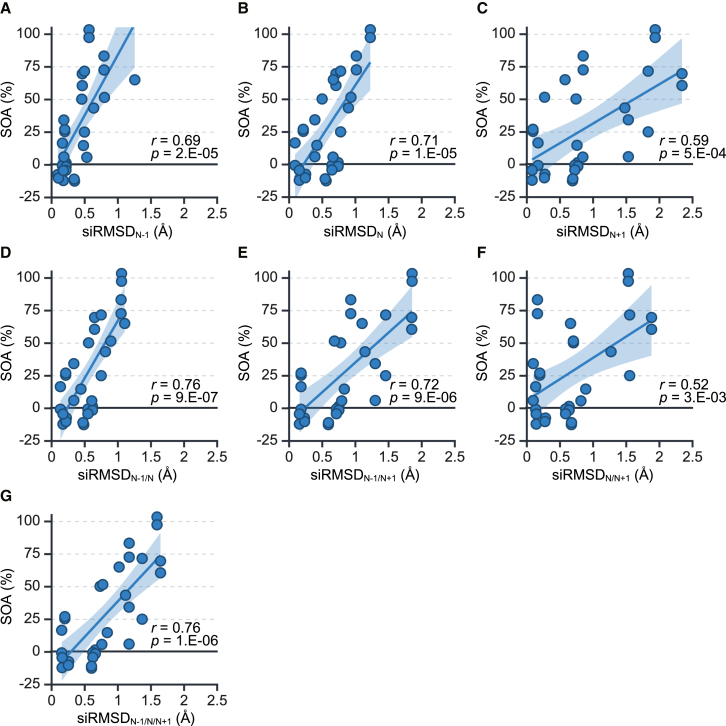
Validation of the siRMSD_N-1/N/N+1_ parameter for predicting off-target activity using siRNAs with different sequences (A–G) Correlation analysis between the SOAs and siRMSD values for siRNAs with 2′-ribose modifications. The blue-shaded areas in represent the 95% confidence intervals.

The structural changes by chemical modifications at positions 2–5 are considered to be essentially same regardless RNA sequences, because the interactions between guide RNA and AGO protein are primarily mediated through phosphate groups and ribose sugars in the backbone of RNA duplex.^43^ Therefore, to validate the correlations at positions 2–5 for siRNAs with different sequences, we tested siRNAs against *clathrin heavy chain* (*CLTC*), *melanocortin 4 receptor* (*MC4R*), and *kinesin family member 23* (*KIF23*) with 2′-ribose modifications. RNAi and off-target activities of these siRNAs were assessed by reporter assays (Figures S22 and S23), and their SOAs were calculated. We then analyzed the correlations between siRMSD_N-1,_ siRMSD_N,_ siRMSD_N+1_, siRMSD_N-1/N_, siRMSD_N/N+1_, siRMSD_N-1/N+1_, and siRMSD_N-1/N/N+1_ values for 2′-ribose modifications (Figures S16B–S21B and 5B) and the SOAs. Among them, siRMSD_N-1/N_ and siRMSD_N-1/N/N+1_ exhibited the highest correlations (*r* = 0.76) with SOAs (Figure 6). Thus, siRMSD_N-1/N/N+1_ may be the good parameter, consistent with the results of siVIM-270 and its variants (Figures S16–S21 and 5).

### Validation of the siRMSD parameter for predicting off-target activity using siRNAs with a different chemical modification

**Figure 7:**
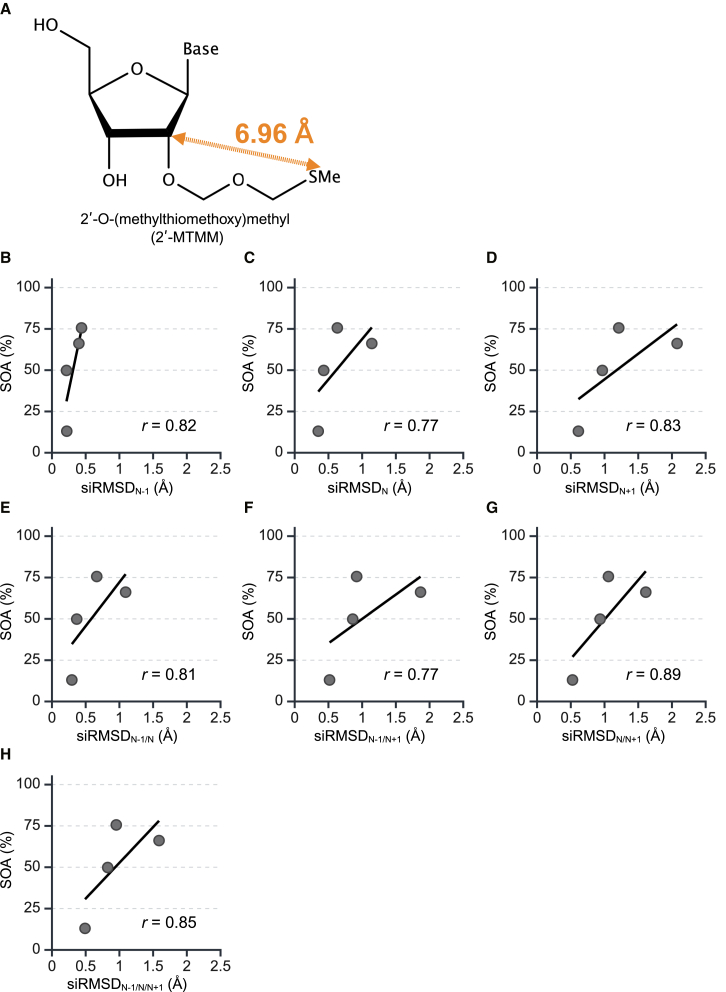
Validation of the siRMSD_N-1/N/N+1_ parameter for predicting off-target activity using siRNAs with 2′-MTMM chemical modification (A) Structure of 2′-MTMM modification, with distance measured by computer-simulated structures (Figure S25). (B–H) Correlation analysis between the SOAs and siRMSD values for siRNAs with 2′-MTMM modifications.

In the next, to validate the accuracy of the siRMSD_N-1/N/N+1_ parameter for siRNAs containing different chemical modification, we used 2′-O-(methylthiomethoxy)methyl (2′-MTMM)^22^ (Figure 7A) which was not used in the original analyses. The steric size of 2′-MTMM modification was the biggest (6.96 Å) among the used modifications in this study. The reporter assays of the MTMM-modified siRNAs (siVIM-270_MTMM_2; siMC4R-490_MTMM_3; siKIF23-430_MTMM_4, _5) were performed to examine their RNAi and off-target activities (Figure S24), and SOAs were calculated. The computational simulations of the siRNA structures with 2′-MTMM modifications at each of positions 2–5 were performed (Figure S25), and their siRMSD values were calculated using the previously described method (Figure S26). The correlations between these siRMSD values and SOAs were then analyzed (Figures 7B–7H). The siRMSD values were high at any positions and any combinations, and siRMSD_N-1/N/N+1_ had a high correlation with SOAs (*r* = 0.85), although the high correlation was also observed with siRMSD_N/N+1_ (*r* = 0.89) (Figure 7). These findings suggest that siRMSD_N-1/N/N+1_ value can provide as a predictive of off-target activity for siRNAs with different types of 2′-ribose modifications.

### Effects of chemical modifications on *T*_*m*_ values and off-target effects

**Figure 8:**
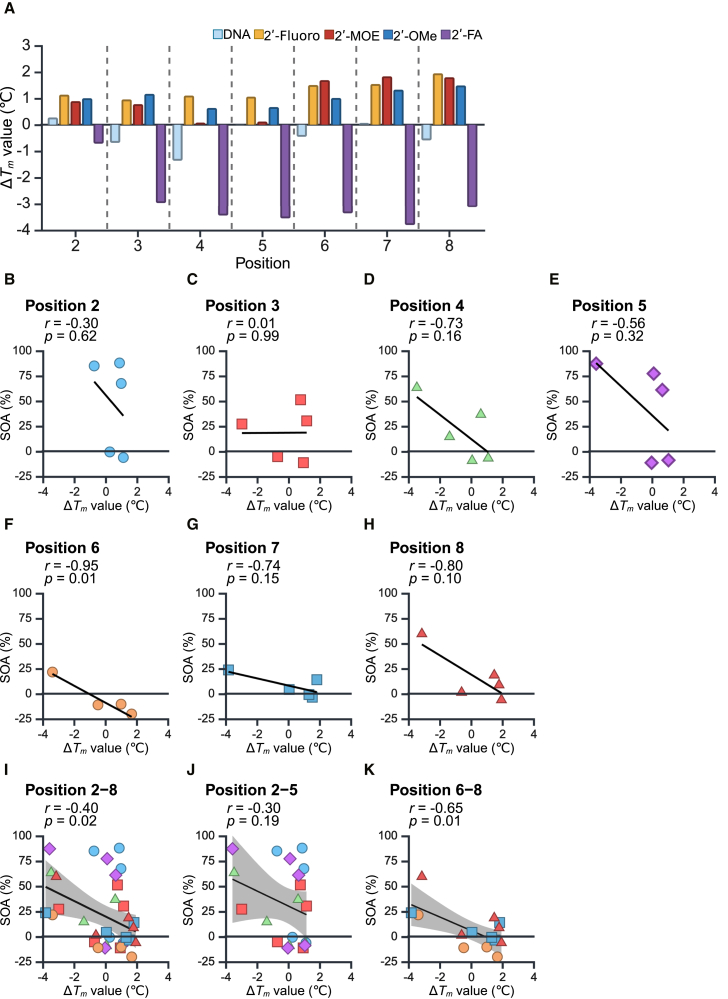
Correlations between melting temperatures and SOAs (A) Changes in melting temperatures (Δ*T*_*m*_) of siVIM-270s with chemical modifications compared to that of unmodified siVIM-270. Horizontal axis indicates the position of chemical modification. Vertical axis, Δ*T*_*m*_ value relative to unmodified siRNA. (B–H) Dot plots of the correlations between *T*_*m*_ values and SOAs at each position 2–8. (I–K) Dot plots of the correlation coefficients between the Δ*T*_*m*_ values and SOAs of nucleotides 2–8 (I), 2–5 (J), and 6–8 (K). The gray-shaded areas in represent the 95% confidence intervals.

It was hypothesized that structural alterations at positions 2–5 resulted in a deviation from the A-form structure, thereby reducing target binding efficiency and minimizing off-target effects. However, the 2′-FA modification at positions 6–8 also suppressed off-target effects, despite the absence of significant structural changes at these positions (Figures 2E and S9–S11). The negligible structural changes at positions 6–8 suggested that an alternative factor may be involved in the observed reduction of off-target effects. One possible explanation is the low thermodynamic stability between the seed region of the siRNA guide strand with 2′-FA modification and the off-target transcript. To explore this point, we measured the melting temperature (*T*_*m*_) values of siVIM-270s with various chemical modifications at different positions within the seed region, and compared their values to that of unmodified siRNA (Figure 8A). Modifications such as 2′-fluoro, 2′-MOE, and 2′-OMe increased the *T*_*m*_ values across all positions in the seed region, although the increase of the *T*_*m*_ value by 2′-MOE modification was less significant at positions 4 and 5. Conversely, the introduction of DNA slightly reduced *T*_*m*_ values at positions 3, 4, 6, and 8, with minimal impact at positions 5 and 7. However, 2′-FA notably decreased *T*_*m*_ values across all positions in the seed region, with reductions of approximately 1°C at position 2 and over 3°C at other positions.

The off-target effects of unmodified siRNAs are positively correlated with the thermodynamic stability of the interaction between the seed region and the off-target transcript, implying that the higher *T*_*m*_ values are associated with the greater off-target effects.^23^ Computational simulations indicated a strong correlation between off-target effects and structural distortions at positions 2–5 (Figure 5K), but this correlation was less significant at positions 6–8 (Figure 5L). However, siRNAs modified with 2′-FA at positions 6–8 had significantly lower *T*_*m*_ values and exhibited reduced off-target effects (Figures 2E and 8A), suggesting that the decreased *T*_*m*_ value due to chemical modification could account for the diminished off-target activity at these positions. To further investigate, we analyzed the correlation between changes in *T*_*m*_ (Δ*T*_*m*_) values caused by chemical modifications at positions 2–8 and the corresponding SOAs (Figures 8B–8H). We found that the Δ*T*_*m*_ values of siRNAs with chemical modifications at positions 6, 7, and 8 were strongly negatively correlated with the SOAs at *r* = −0.95, −0.74, and −0.80, respectively (Figures 8F–8H). While the Δ*T*_*m*_ values of siRNAs with chemical modifications at positions 2 to 5 were relatively lower (*r* = −0.30, 0.01, −0.73, and −0.56, respectively) (Figures 8B–8E). Therefore, the correlations between Δ*T*_*m*_ values and off-target activities for the entire seed region (positions 2–8), positions 2–5, and positions 6–8, were calculated separately (Figures 8I–8K). Although the correlation coefficients for positions 2–8 and 2–5 were weak at −0.40 and −0.30, respectively (Figures 8I and 8J), that for positions 6–8 showed the highest correlation coefficient at −0.65 (Figure 8K). Specifically, the 2′-FA modification at positions 6–8 may reduce off-target effects by significantly lowering *T*_*m*_ values, in addition to the steric hindrance observed at positions 2–5. Thus, while modification types II (2′-MOE and 2′-OMe) and III (2′-FA) can reduce off-target effects by inducing structural distortions at positions 2–5, the type III modification also decreases off-target effects by reducing thermodynamic stability at positions 6–8 (Figure 2G).

## Discussion

In this study, we revealed the structural impact of 2′-ribose modifications on RNAi and off-target effects of siRNAs. The larger-sized modifications at 2′-ribose, such as 2′-MOE (5.92 Å), 2′-FA (3.50 Å), and 2′-MMTM (6.96 Å), were revealed to decrease the RNAi activities at position 2 (Figures 1, 2D, 7A, S23A, S23B, and S24A). In contrast, smaller modifications like DNA (1.10 Å), 2′-fluoro (1.39 Å), and 2′-OMe (3.26 Å) did not affect RNAi activities. These results suggested that RNAi activity is strongly affected by the size of the 2′-ribose modification at position 2. The nucleotide at position 2 exhibited the largest siRMSD values (Figure 5B), likely due to the nucleotide at position 1 is strongly fixed within the pocket of AGO2 MID domain through multiple hydrogen bonds.^49^ This strong anchoring may result in significant structural changes of nucleotides 2 and 3 when large-sized modifications are introduced into nucleotide at position 2. During the initial target recognition process, the AGO2 protein primarily utilizes nucleotides at positions 2, 3, and 4 of the guide RNA to find out complementary sequences in the target mRNA.^50^ Consequently, substantial structural changes at positions 2 and 3 may disrupt this early target recognition step, leading to decreased RNAi activity. The siRMSD_N-1/N/N+1_ values of siRNA with 2′-MOE, 2′-FA, and 2′-MMTM at position 2 were high at 1.65, 1.60, and 1.60, respectively (Figures 5B and S26G). However, the values for 2′-OMe, DNA, and 2′-fluoro were low at 1.37, 0.67, and 0.63, respectively. These results suggested that the size of modifications at position 2 have strong correlation with RNAi activities. Furthermore, our experiments demonstrated that this effect was independent of phosphorylation at 5′ end of siRNA (Figure S12). Even in the presence of a 5′-phosphate, siRNAs with a 2′-MOE modification at position 2 exhibited reduced RNAi activity, suggesting that strong steric hindrance plays a dominant role over potential effects of phosphorylation impairment.

Additionally, 2′-OMe, 2′-FA, and 2′-MMTM at nucleotides 3–5, as well as 2′-MOE at nucleotides 3 and 5, reduced off-target effects without significantly affecting RNAi activity. From the structural simulation, it was found that they can disrupt the pre-organized A-form structure of siRNA on AGO2, even when each modification was introduced one by one into each nucleotide, which may increase the entropic cost of seed pairing and hinder efficient target binding. This may explain why these modifications reduce off-target effects, as off-target effects are primarily induced by base-pairing between the siRNA seed region and the target mRNA. However, the previous analyses revealed that the base stacking of guide RNA on AGO2 is interrupted by a kink between nucleotides 6 and 7, and this kink is promoted by the helix-7 domain of AGO2.^39^^,^^42^^,^^43^ Thus, helix-7 creates a steric barrier to base-pairing beyond nucleotide 5. Furthermore, the single-molecule analyses revealed that mismatches in nucleotides 2–5 of guide RNA are far more sensitive than those in nucleotides 6–8.^50^^,^^51^ Therefore, it is suggested that disruption of base-pairing with target mRNA within nucleotides 3–5 can be compensated by the base-pairing from nucleotides following 6 for RNAi activity.

We also demonstrated that the siRMSD_N-1/N/N+1_ value, which represents the sum of the averaged squared distances of all atoms in the nucleobases at N−1, N, and N+1 in the modified siRNA, is a highly suitable predictor of off-target effects. This predictor had a high correlation coefficient with off-target activity at positions 2–5, suggesting the importance of maintaining the A-form at these positions.^40^^,^^42^ Furthermore, validation studies demonstrated that the siRMSD_N-1/N/N+1_ consistently showed strong correlations with off-target effects for the siRNAs with different sequences and with a different type of 2′-ribose modification, confirming its robustness as a predictive tool. Compared to thermodynamic parameters such as Δ*G* and Δ*T*_*m*_, which represent the stability of seed-target duplex, siRMSD provides a complementary structural perspective by quantifying local RNA distortions on AGO2. Unlike machine learning-based predictors, siRMSD is grounded in first-principles structural modeling, eliminating the need for training data and enabling direct application to novel chemical modifications. This is particularly relevant given that machine learning models for siRNA efficiency are often constrained by the limited availability of high-quality training data.^52^ Therefore, this parameter could be used as a new parameter to predict the reduction of off-target effects due to chemical modifications.

At positions 6–8, the reductions in off-target effects were not related to structural distortions but to thermodynamic stability (as indicated by *T*_*m*_ value). When superimposing the structures of guide RNAs and AGO2 proteins from the PDB, nucleotides at positions 6–8 exhibited various RNA structures, suggesting that these nucleotides might be acceptable to form flexible structures on AGO2. Therefore, the thermodynamic stability had a marked influence on off-target effects but the structural distortion did not. Notably, 2′-FA was particularly valid in significantly reducing off-target effects even with a single modification by lowering *T*_*m*_ values. These results may explain why glycol nucleic acid (GNA) and TNA, which decrease thermodynamic stability, reduce off-target effects when introduced at position 7.^44^^,^^53^ In contrast, introducing DNA one by one into the seed region slightly decreased the *T*_*m*_ value (Figure 8A), yet had the negligible effect on off-target effect (Figure 2E). However, the sequential modifications in the seed region of DNAs have the apparent effects on reducing off-target effects,^20^^,^^31^ probably decreasing *T*_*m*_ values sufficiently.

The structural distortions at positions 2–5 and the decreased thermodynamic stability at positions 6–8 may primarily influence -off-target effects rather than RNAi activity. This distinction arises because off-target effects are mainly driven by base-pairing between the siRNA seed region (nucleotides 2–8) and unintended target mRNAs, relying on only seven nucleotides for interaction. Consequently, structural perturbations or reduced binding stability in this region significantly affect off-target interactions. In contrast, RNAi activity depends on sequence complementarity across the guide strand with the intended target mRNA, allowing for compensatory base-pairing interactions beyond the seed region. As a result, while modifications at positions 2–8 can reduce off-target effects, their impact on RNAi activity is less pronounced, as the extensive base-pairing in fully complementary target sites can offset these structural or thermodynamic changes.

Although this study was conducted exclusively in HeLa cells, our previous findings showed that siRNAs exhibited essentially comparable RNAi activity in other human cell lines.^54^ Furthermore, a previous study reported that the effects of chemical modifications on siRNA activity were similar across different mammalian cell types,^55^ probably because AGO2 function is largely conserved across cell types. Thus, the effects of 2′-ribose modifications on RNAi and off-target activity observed in HeLa cells are expected to be similar in other mammalian cells.

This study could provide crucial insights into chemical modifications for the rational design of siRNAs for therapeutics. Furthermore, it was demonstrated that the chemical modifications can be classified into three types I, II, and III based on their structural and thermodynamic properties. This may offer a valuable framework for selecting modifications tailored to therapeutic needs. In particular, while 2′-ribose modifications have primarily been employed to enhance nuclease stability in systemic circulation, our findings suggest that their strategic placement can also effectively suppress off-target effects, thereby optimizing siRNAs for therapeutic applications. However, further studies are needed to elucidate how multiple modifications cooperatively influence off-target effects. In addition, it should be noted that all siRNAs used in this study were classified as class I, which is a siRNA category of highly functional and off-target-reduced siRNAs defined in our previous study.^56^ They have common features, possessing A/U at the 5′-end of guide strand, G/C at the 5′-end of passenger strand, and at least four A/U nucleotides in a 7-nucleotide region of the 5′-terminal of the guide strand. Further study is necessary to investigate the possibility for applying the same strategy to other classes of siRNAs.

DFT-based models represent idealized, static conformations and may not fully capture the structural flexibility of siRNAs under physiological conditions. These simulations are intended to illustrate relative distortions caused by chemical modifications, not to replicate actual intracellular geometries. Local siRNA structures may vary depending on factors, such as RNA-binding proteins, ionic conditions, and temperature.

## Materials and methods

### Chemical synthesis of siRNA duplexes

RNA oligonucleotides corresponding to the guide and passenger strands of each unmodified siRNA and siRNA with DNA, 2′-fluoro, 2′-MOE, or 2′-OMe modification were chemically synthesized (Shanghai Gene Pharma, Shanghai, China; Gene Design, Osaka, Japan) and subsequently annealed to form siRNA duplexes. The synthesis of siRNA containing 2′-FA modifications has been previously described.^34^ The procedure to synthesize siRNA containing 2′-O-(methylthiomethoxy)methyl (2′-MTMM) have been previously described.^22^ siRNA sequences used in this study were summarized in Table S2. The number following each siRNA name indicates the nucleotide position in each target gene corresponding to the 3′ end of siRNA guide strand, and the following number indicates the position with DNA, 2′-fluoro, 2′-MOE, 2′-OMe, or 2′-FA modification.

### Cell culture

Human HeLa cells were cultured in Dulbecco’s Modified Eagle’s Medium (FUJIFILM Wako, Osaka, Japan) containing 10% fetal bovine serum (Gibco Life Technologies, Paisley, UK) and 1% penicillin-streptomycin solution (FUJIFILM Wako) at 37°C with 5% CO_2_.

### Construction of luciferase reporter constructs with completely and seed-matched-sequences of siRNAs

All the reporter plasmids used in this study were derived from psiCHECK-1 (Promega, Madison, WI, USA). Oligonucleotides with a completelymatched (CM) sequence and three tandem repeats of seed-matched (SM) sequence of each siRNA with Xhol/EcoRI sticky ends were chemically synthesized (Sigma-Aldrich, Japan). Each of these oligonucleotides was inserted into the 3′ UTR region of *Renilla luciferase* (*luc*) gene in psiCHECK-1 by digestion with the same restriction enzymes. Sequences of oligonucleotides with CM or SM sequences are summarized in Table S3.

### RNA silencing activity assay

RNAi and off-target activities were measured using a luciferase reporter assay. A HeLa cell suspension (1.0 × 10^5^ cells/mL) was inoculated into a well of 24-well culture plates 1 day before transfection. Cells were simultaneously transfected with 0.005, 0.05, 0.5, or 5 nM of each siRNA, 0.1 μg of pGL3-control vector (Promega) encoding the firefly luciferase gene, and 0.01 μg of each psiCHECK construct with CM or SM target sequence using Lipofectamine 2000 (Thermo Fisher Scientific, Waltham, MA, USA). siGY441 with unrelated sequence was used as a control siRNA (siCont). At 24 h after transfection, the cells were lysed with 1 × passive lysis buffer (Promega). Luciferase activity was measured using the Dual-Luciferase Reporter Assay System (Promega) and GloMax Discover Microplate Reader (Promega), and the *Renilla* luciferase activity normalized by firefly luciferase activity (*Renilla* luciferase activity/firefly luciferase activity) was calculated. The half maximal inhibitory concentrations (IC_50_s) of siRNAs were estimated using Equation 2.

(Equation 2) $${\text{IC}}_{50}=10^{\log_{10}\left(\frac{A}{B}\right)\times \frac{50-C}{D-C}+\log_{10}\left(B\right)}$$

A: Concentration when inhibitory efficiency is lower than 50%,

B: Concentration when inhibitory efficiency is higher than 50%,

C: Inhibitory efficiency in B,

D: Inhibitory efficiency in A.

### Microarray analysis

A HeLa cell suspension (1.0 × 10^5^ cells/mL) was inoculated into a well of 24-well culture plate 16 h prior to transfection. Cells were transfected with 50 nM of each siRNA using Lipofectamine 2000 (Thermo Fisher Scientific, Waltham, MA, USA) according to the manufacturer’s instructions. Twenty-four hours after transfection, cells were harvested from two wells for each sample. Total RNA was purified using the RNeasy Mini Kit (QIAGEN, Hilden, Germany), and the quality of the total RNA was confirmed using a NanoDrop 2000 spectrophotometer (Thermo Fisher Scientific, Waltham, MA, USA) and a Bioanalyzer (Agilent Technologies, Santa Clara, CA, USA).

cDNA and Cy3-labeled RNA were synthesized using the Quick Amp Labeling Kit for One-Color (Agilent Technologies). The Cy3-labeled RNAs were fragmented using the Gene Expression Hybridization Kit (Agilent Technologies) and hybridized to a SurePrint G3 Human GE Microarray (version 3, 8 × 60K) (Agilent Technologies) at 65°C for 17 h. After washing, the microarray slide was scanned by a DNA Microarray Scanner (Agilent Technologies), and the signals were quantified using Feature Extraction software version 10.5.1.1 (Agilent Technologies).

For analysis, the data satisfying the following conditions were selected: ControlType = 0, gIsPosAndSignif = 1, gIsFeatNonUnifOL = 0, gIsWellAboveBG = 1, gIsSaturated = 0, gIsFeatPopnOL = 0, and SystematicName = NM_Identifier. As a result, total of 11,919 transcripts were used for analysis. Among them, 1,185 were off-target transcripts with sequences complementary to the seed region of each siRNA in their 3′ UTRs. RNA from mock-transfected cells, treated with the transfection reagent without siRNA, served as a control. The signal intensities of the transcripts across all samples were normalized using quantile normalization.^57^^,^^58^ Results were presented in MA plots and cumulative distribution plots.

### RT-qPCR

Total RNAs used for microarray analysis were also applied for RT-qPCR. Total RNA isolated from the cells subjected to mock transfection, where the transfection reagent was applied without siRNA, was used as the control. An aliquot of total RNA (1 μg) from each sample was reverse transcribed using the high-capacity cDNA reverse transcription kit (Applied Biosystems, Foster City, CA, USA) according to the manufacturer’s instructions. The RT-qPCR was performed using the KAPA SYBR Fast qPCR Kit (NIPPON Genetics) on a QuantStudio 3 real-time PCR system (Applied Biosystems) using the ΔΔCt method. The expression levels of the target genes were normalized to the endogenous reference gene, *glyceraldehyde-3-phosphate dehydrogenase* (*GAPDH*). Subsequent normalization was carried out against the mock-transfected samples. The sequences of the primer sets used are shown in Table S4.

### Computational prediction of the structure of modified RNA

The Cartesian coordinates of the crystal structures (PDB IDs: 4W5O and 4F3T) were used as the starting points for positioning initial geometry of RNAs on the AGO2 protein. We considered amino acids of the AGO2 protein within 4 Å of RNAs for potential interactions. These amino acids had their main chains replaced with methyl groups at the atoms bonded to Cα atom. And we introduced DNA, 2′-fluoro, 2′-MOE, 2′-OMe, or 2′-FA modification at all positions in the siRNA seed region. Full geometry optimization was performed at the theoretical level of ωB97X-D/6-31G(d) by keeping the Cα atoms of amino acids fixed, except for those at positions 6 and 7 (Met 364 (M364) and Ile 365 (I365). The RNAs and amino acids used in the calculation for each position and PDB IDs listed on Table S1. The resulting energies and Cartesian coordinates are listed in Tables S5 and S6. All models of chemical structures used in the geometry optimization are displayed in Figure S4.

### siRMSD calculation

RMSD calculations were performed to quantify the structural distortion of siRNA with chemical modifications compared to the unmodified siRNA on AGO2 protein, and named as siRMSD. The siRMSD was calculated using Equation 3.

(Equation 3) $$\text{siRMSD}\;\left(Å\right)=\sqrt{\frac{1}{K}\;\sum_{i=1}^{k}{\delta_{i}}^{2}}$$

where *δ*_*i*_ represents the distance (Å) between a specific atom *i* in the chemically modified RNA and the corresponding atom in the unmodified RNA structure. *K* represents the total number of equivalent atoms considered in the calculation. The coordinates of the atoms (excluding hydrogen atoms) in the RNA nucleobases, obtained by computational simulation results, were used for the calculations. The calculation was performed using the “rmsd” Python library (version 1.5.1), which is available on GitHub (https://github.com/charnley/rmsd).

### Measurement of melting temperature

For measuring the melting temperature (*T*_*m*_) value of each siRNA, we prepared and mixed the following materials: RNA and its complementary RNA, both at a final concentration of 5 μM, in a total volume of 100 μL D-PBS (FUJIFILM Wako, Osaka, Japan). The mixture was thoroughly mixed to ensure homogeneity. The *T*_*m*_ value was measured by setting the program to change the temperature from 90°C to 30°C and back to 90°C at a rate of 0.5°C per minute, while measuring the absorbance at 260 nm using a V-650 spectrophotometer (Jasco, Japan). The absorbance change with temperature was automatically drawn, and the *T*_*m*_ value was consequently calculated based on the second derivative of the curve. The measurement results are shown as Δ*T*_*m*_, the difference from the *T*_*m*_ value of unmodified siRNA targeting the human *vimentin* gene (siVIM-270) (74.5°C).

## Data availability

All data needed to evaluate the conclusions in the paper are present in the paper and/or the Supplementary Materials, including dose-dependent luciferase reporter assays, microarray, RT-qPCR, and additional computational simulation data; sequences of siRNA, reporter construct and primers; amino acid lists considered in computational simulation, total energy, and coordinates. The microarray data generated in this study have been deposited in the NCBI GEO database (accession numbers: GSE278438 and GSE278439).

## Acknowledgments

Part of the simulations using the Gaussian 16 package^59^ were carried out at the Center for Quantum Life Sciences and the Research Center for Computational Science, Okazaki National Research Institutes. We thank M. Aida and D. Akase at Hiroshima University for their guidance and advice regarding the computational simulations. The English in this document has been checked by at least two professional editors, both native speakers of English. This work was supported by the 10.13039/501100001700Ministry of Education, Culture, Sports, Science and Technology of Japan (grant no 21H02465), the Kurata grant awarded by the 10.13039/501100012009Hitachi Global Foundation, and the 10.13039/100009619Japan Agency for Medical Research and Development (10.13039/100009619AMED, grant no 21ae0121032h0001 and 25ae0121057s0702), all attributed to K.U.-T.

## Author contributions

S.A., Y. Kobayashi, and K.U.-T. initially designed the study, discussed the experimental methods, results, and findings. K.N., Y. Kimura, and H.A. synthesized the 2′-formamido-modified siRNAs used in the experiments. S.A. performed the experiments and computational simulations. S.A. and K.N. measured the thermodynamic stabilities of the siRNAs. The initial draft of the manuscript was written by S.A. and Y. Kobayashi, and revised and completed by S.A. and K.U.-T. All authors read and approved the final manuscript.

## Declaration of interests

All other authors declare they have no competing interests.

## Supplemental information

Document S1. Figures S1–S26 and Tables S1, S3 and S4

Table S2. Nucleotide sequences of siRNAs

Table S5. Total energies of optimized structures

Table S6. Cartesian coordinates of optimized structures

Document S2. Article plus supplemental information
