# Supplementary material for: An siRMSD parameter of structural distortion induced by chemical modification is predictive of the off-target effect of siRNA
Source: Mol Ther Nucleic Acids. 2025 Sep 16;36(4):102693. doi: 10.1016/j.omtn.2025.102693 (PMC12744844; doi:10.1016/j.omtn.2025.102693)
Supplement: Document S1. Figures S1–S26 and Tables S1, S3 and S4 [file mmc1.pdf]

**OMTN, Volume *36***

## Supplemental information

# An siRMSD parameter of structural distortion induced by chemical modification is predictive of the off-target effect of siRNA

**Seongjin An, Kohei Nomura, Yoshiaki Kobayashi, Yasuaki Kimura, Hiroshi Abe, and Kumiko Ui-Tei**

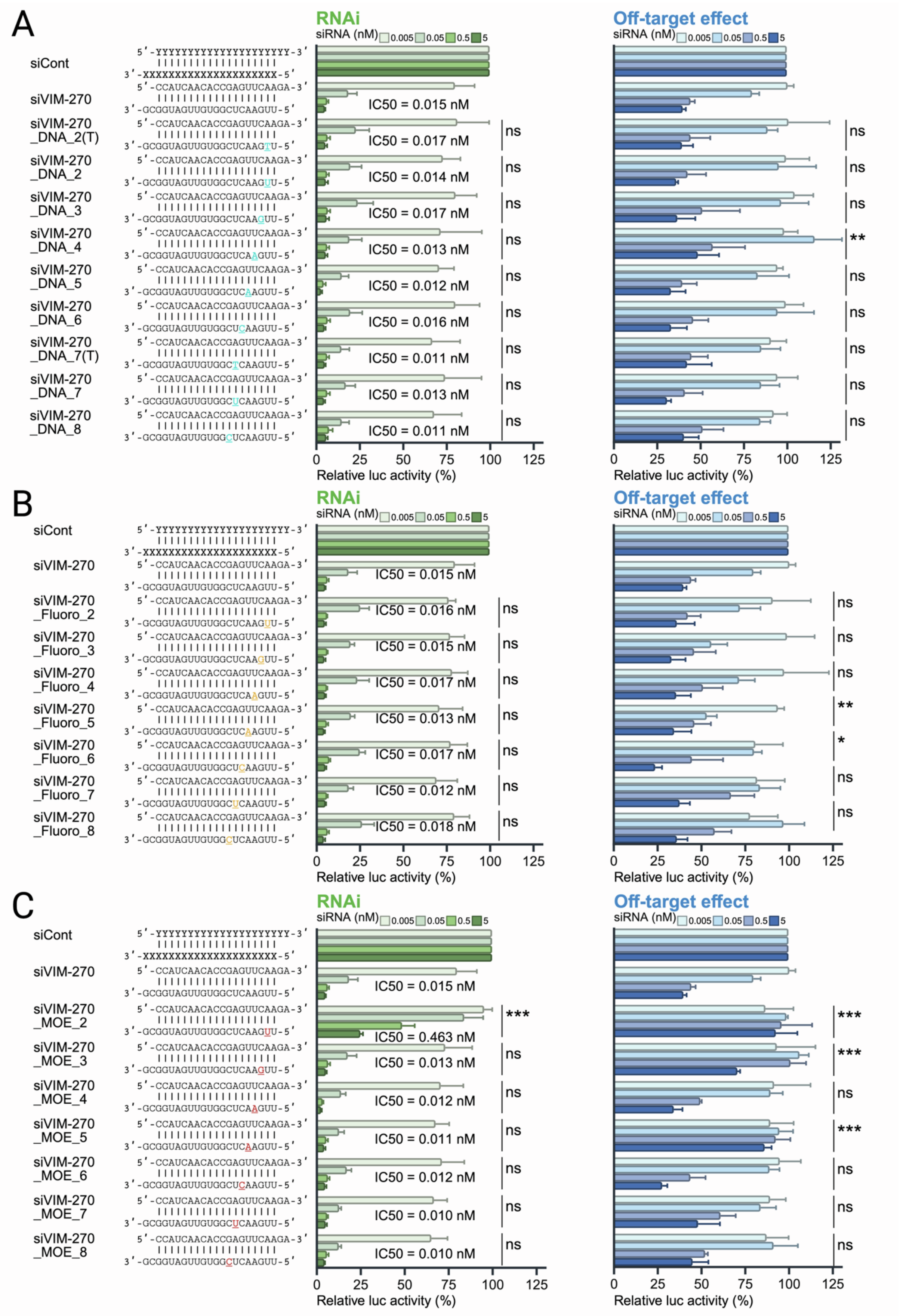

A
B
C
RNAi
Off-target effect
siRNA (nM) 0.005 0.05 0.5 5
siCont
siVIM-270
siVIM-270_DNA_2(T)
siVIM-270_DNA_2
siVIM-270_DNA_3
siVIM-270_DNA_4
siVIM-270_DNA_5
siVIM-270_DNA_6
siVIM-270_DNA_7(T)
siVIM-270_DNA_7
siVIM-270_DNA_8
siVIM-270_Fluoro_2
siVIM-270_Fluoro_3
siVIM-270_Fluoro_4
siVIM-270_Fluoro_5
siVIM-270_Fluoro_6
siVIM-270_Fluoro_7
siVIM-270_Fluoro_8
siVIM-270_MOE_2
siVIM-270_MOE_3
siVIM-270_MOE_4
siVIM-270_MOE_5
siVIM-270_MOE_6
siVIM-270_MOE_7
siVIM-270_MOE_8
5′-CCAUCAACACCGAGUUCAAGA-3′
3′-GCGGUAGUUGUGGCUCAAGUU-5′
Relative luc activity (%)

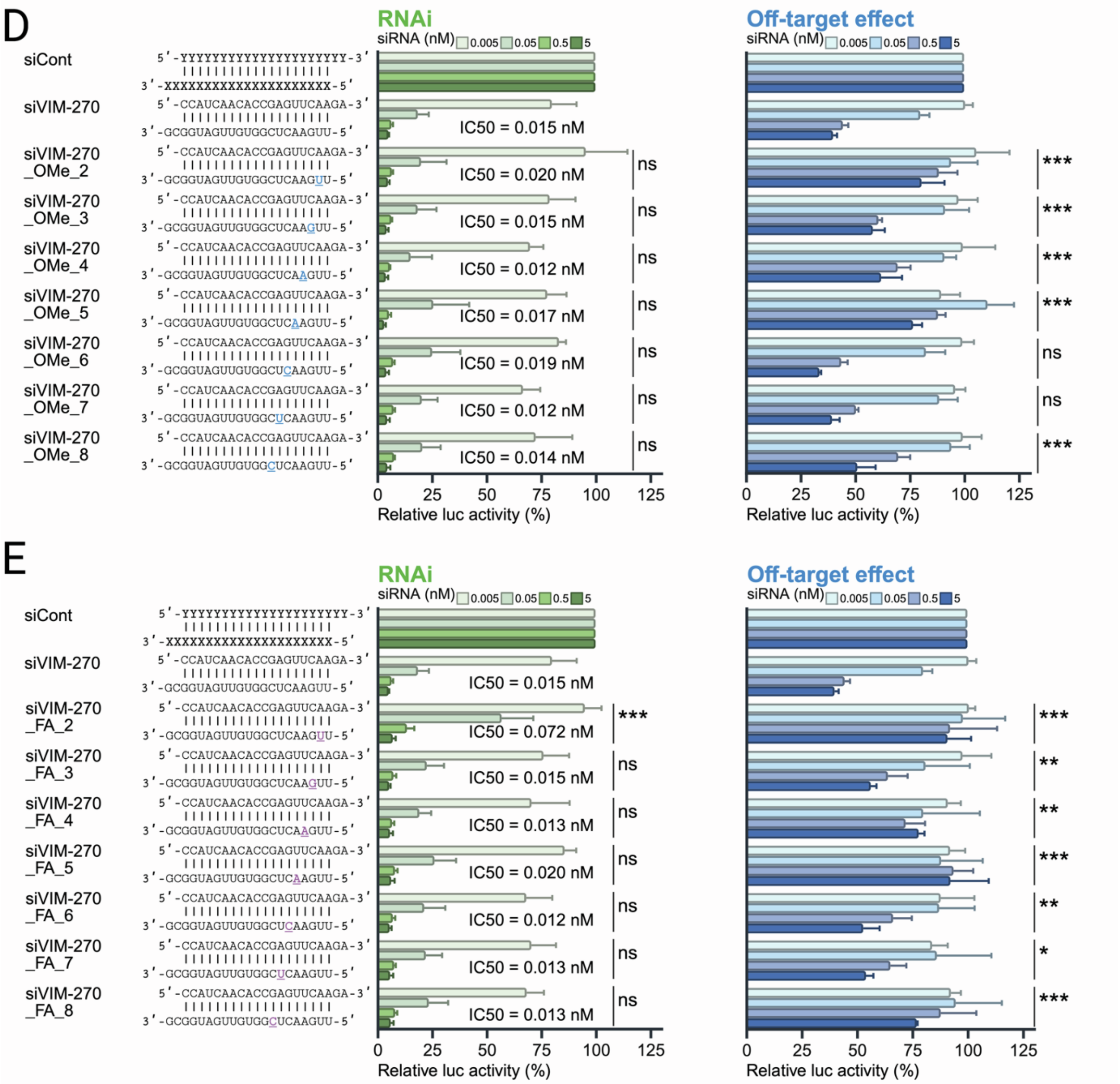


**Figure S1. Dose dependent RNAi and off-target activities of unmodified and modified siVIM-270s.**

The upper RNA strand represents the passenger strand, and the lower represents the guide strand. Green graphs depict RNAi activities, while blue graphs indicate off-target activities. In the siRNA sequence, the colored characters indicate the positions of nucleotides with modifications: light blue, DNA (**A**); yellow, 2'-Fluoro (**B**); orange, 2'-MOE (**C**); blue, 2'-OMe (**D**); and purple, 2'-FA (**E**). Numbers following the names of chemical modifications denote the positions where the modifications were introduced. RNAi/off-target activity of siRNA targeting unrelated gene served as the control siRNA (siCont), and its value was set as 100%. *p*-values were calculated using a two-way ANOVA. Significant levels were indicated as follows: $*p<0.05$, $**p<0.01$, $***p<0.001$. Each experiment was conducted in triplicate.

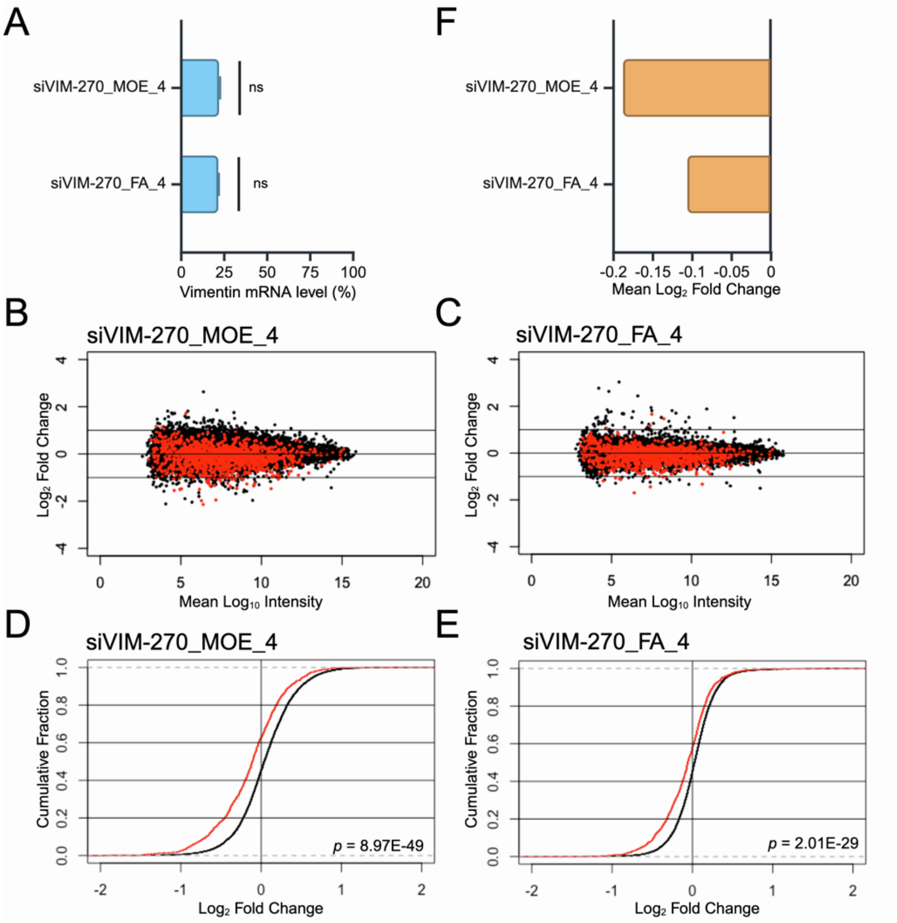


**Figure S2. Expression levels of *vimentin* mRNA and off-target mRNAs after transfection of siVIM-270 with chemical modifications at position 4.**

(**A**) mRNA levels of *vimentin* after transfection of siVIM-270_MOE_4 or siVIM-270_FA_4 compared to mock, as determined by qRT-PCR. One-way ANOVA relative to unmodified siVIM-270 were performed. **p* < 0.05, ***p* < 0.01, ****p* < 0.001. (**B**, **C**) MA plots of microarray results. Vertical axis, $\log_2$ fold changes; horizontal axis, average $\log_{10}$ signal intensities of transcripts in cells transfected with siVIM-270_MOE_4 (**B**) or siVIM-270_FA_4 (**C**) versus mock-transfected cells. Red dots, 1185 off-target transcripts with the siVIM-270 SM sequence in their 3'-UTRs; black dots, 11,919 non-off-target transcripts without the SM sequence. (**D**, **E**) Cumulative distributions of $\log_2$ fold changes (horizontal axis) against cumulative fractions (vertical axis) for off-target (red lines) and non-off-target (black lines) transcripts in cells transfected with siVIM-270_MOE_4 (**D**) or siVIM-270_FA_4 (**E**). *p*-values were calculated by Wilcoxon rank-sum test. (**F**) Seed-dependent off-target effects measured using microarrays were shown as mean $\log_2$ fold changes.

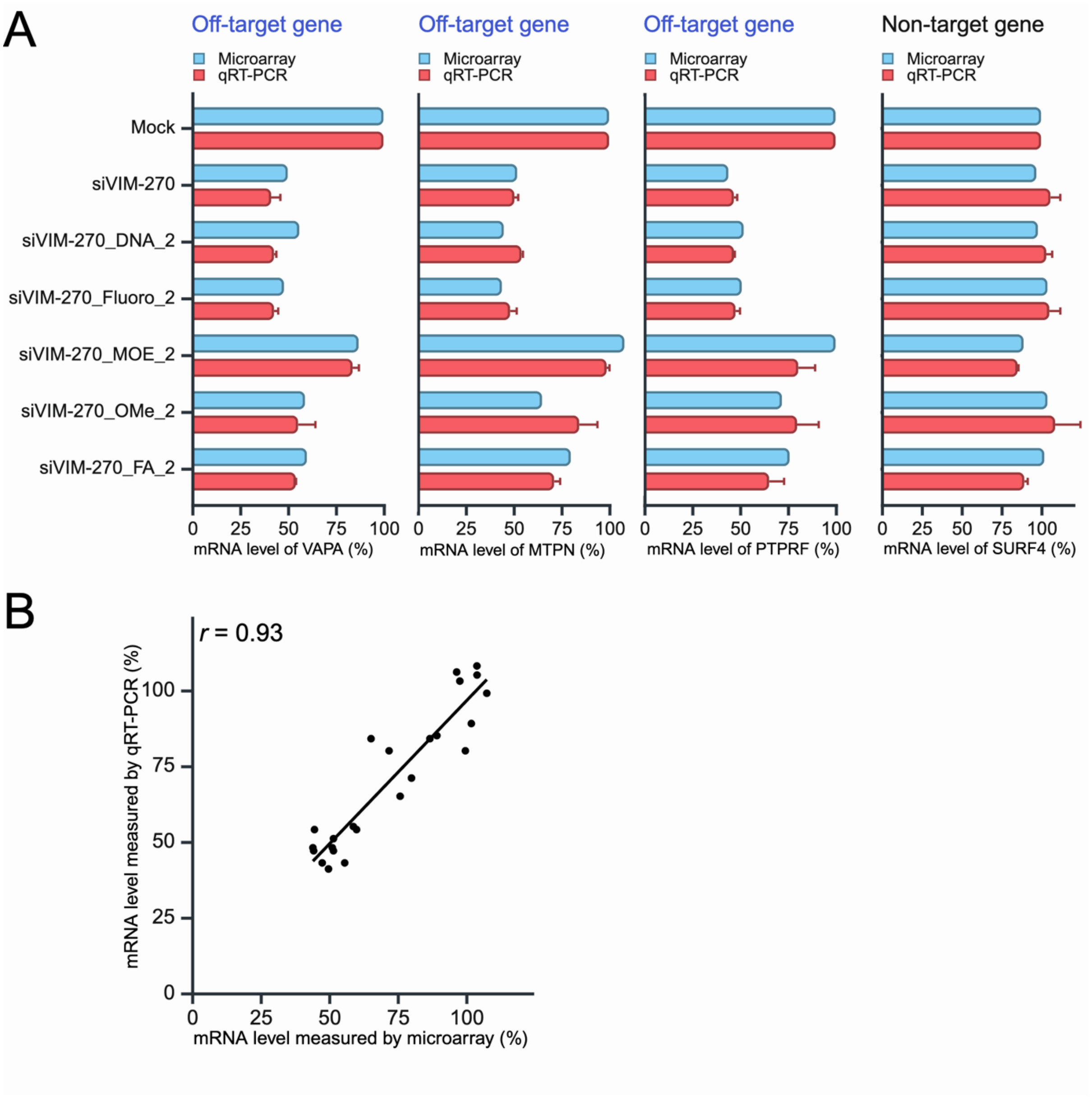


**Figure S3. Comparison of gene expression levels analyzed by microarray and qRT-PCR.** (**A**) Comparisons of microarray and qRT-PCR data of off-target and non-target genes. As off-target genes, *VAPA*, *MTPN*, *PTPRF* were used. As a non-target gene, *SURF4* was used. (**B**) Comparative analysis of gene expression levels of *VAPA*, *MTPN*, *PTPRF*, *SURF4*. Their correlation coefficient was high at 0.93.

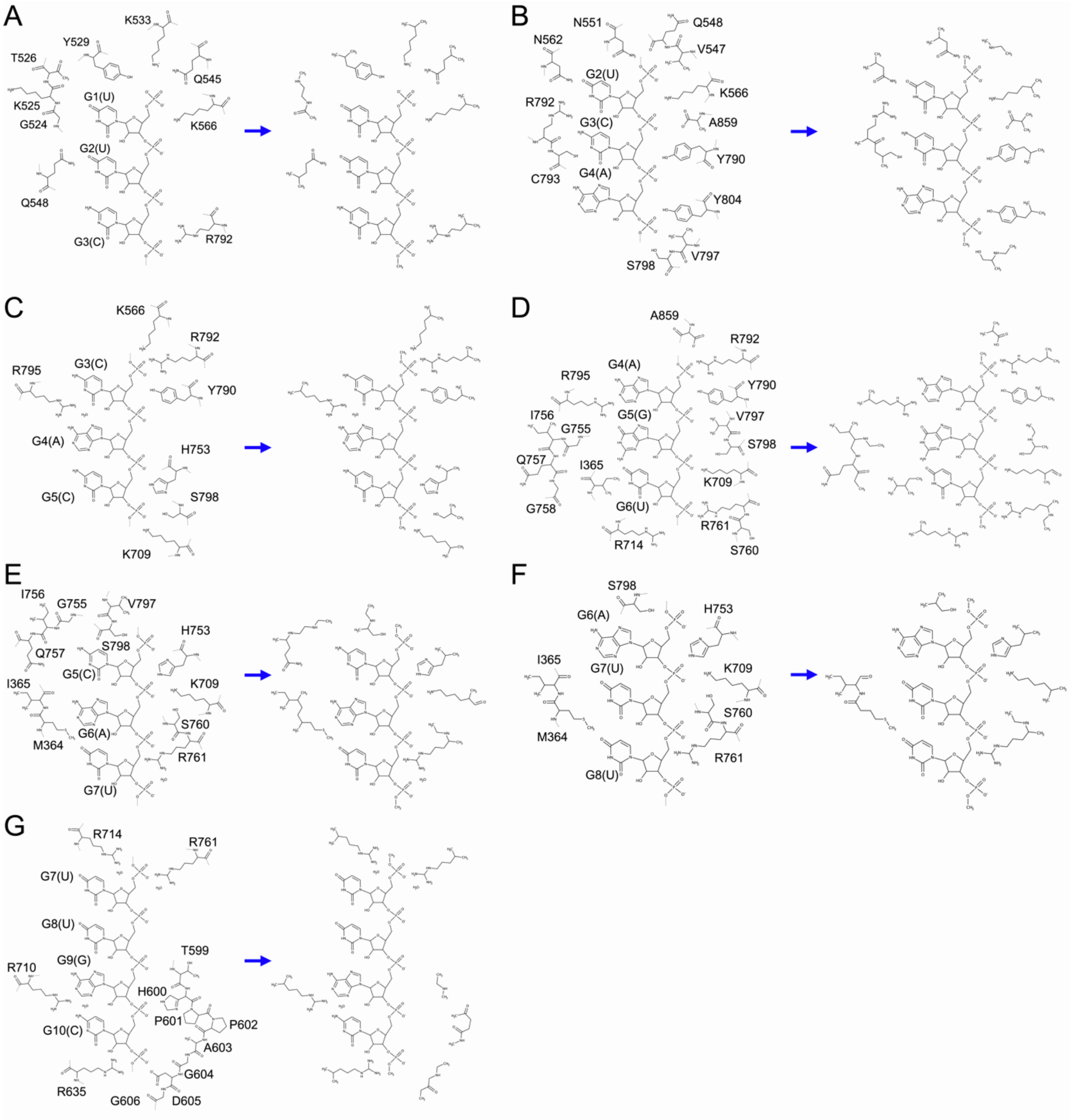


**Figure S4. Geometry optimization of the guide RNA and amino acids of AGO2 protein.**
The optimization was performed at the ωB97X-D/6-31G(d) level fixed with the alpha carbons of the amino acids on the surface of AGO2. Three or four RNA segments from the 5' end of the guide RNA were included in each figure as follows: (**A**) nucleotides 1-3, (**B**) 2-4, (**C**) 3-5, (**D**) 4-6, (**E**) 5-7, (**F**) 6-8, and (**G**) 7-10 for the simulation of structures with chemical modification in the nucleotide 2, 3, 4, 5, 6, 7, and 8, respectively. The original structures of (**A**), (**B**), (**C**), (**E**), (**F**) and (**G**) were derived from PDB ID 4W5O, and (**D**) was from PDB ID 4F3T. Amino acid residues of AGO2 protein considered in the calculations were also included in each figure. Except for the terminal nucleotide at position 1, both ends of the nucleotides were capped with $CH_3$ groups. To enhance computational prediction efficiency, only the amino acids interacting with nucleotides (< 4Å) were retained. The main chain portions of the amino acid residues were replaced with $CH_2$/$CH_3$ groups.

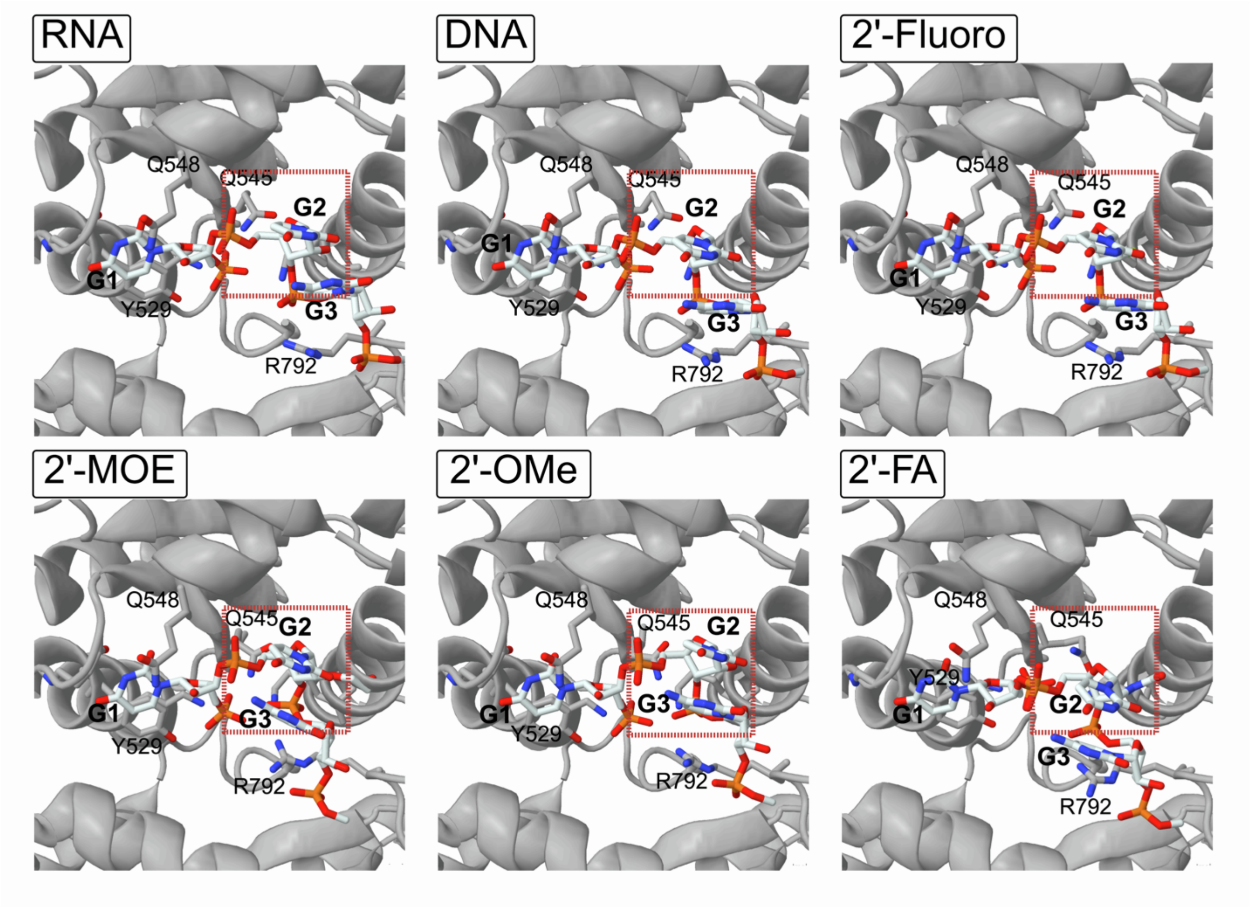


**Figure S5. Computational simulations of nucleotides at position 2.**

As the original RNA structure, Figure S4A, including the nucleotides 1, 2, and 3, was used, and DNA, 2'-Fluoro, 2'-MOE, 2'-OMe, or 2'-FA was introduced at position 2. The amino acid residues of the AGO protein used in these calculations were G524, K525, T526, Y529, K533, Q545, Q548, K566, and R792 (Table S1). The carbon atoms on AGO protein were shown in gray, while the carbon atoms of the guide RNA were shown in white. Oxygen atoms, red. Phosphorus atoms, orange. Nitrogen atoms, blue. Regions expected to be affected by chemical modifications were highlighted with red dashed squares. Enlarged structures are shown in Figure 4A.

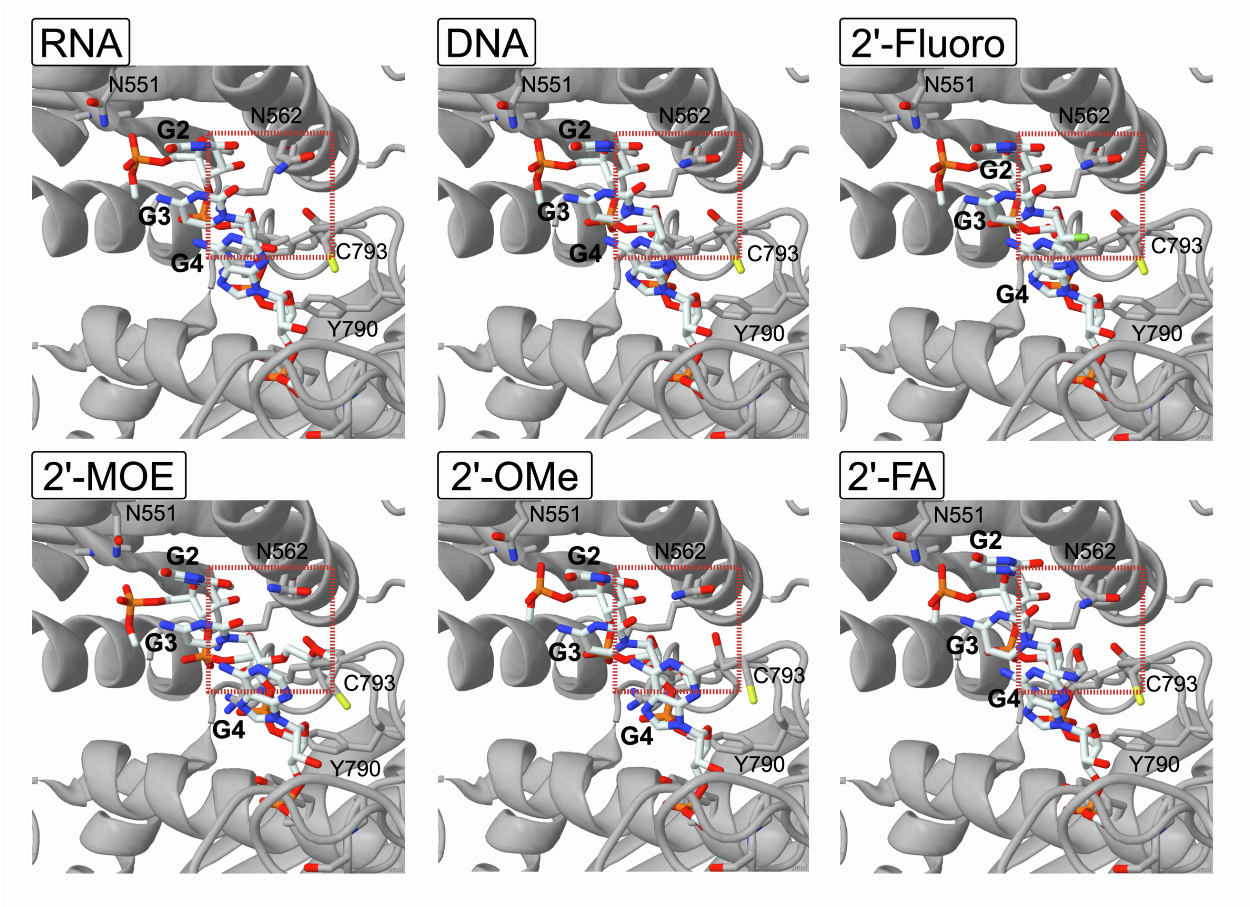


**Figure S6. Computational simulations of nucleotides at position 3.**

As the original RNA structure, Figure S4B, including the nucleotides 2, 3, and 4, was used, and DNA, 2'-Fluoro, 2'-MOE, 2'-OMe, or 2'-FA was introduced at position 3. The amino acid residues of the AGO protein used in these calculations were V547, Q548, N551, N562, K566, Y790, R792, C793, V797, S798, Y804, and A859 (Table S1). The carbon atoms on AGO protein were shown in gray, while the carbon atoms of the guide RNA were shown in white. Oxygen atoms, red. Phosphorus atoms, orange. Nitrogen atoms, blue. Regions expected to be affected by chemical modifications were highlighted with red dashed squares. Enlarged structures are shown in Figure 4B.

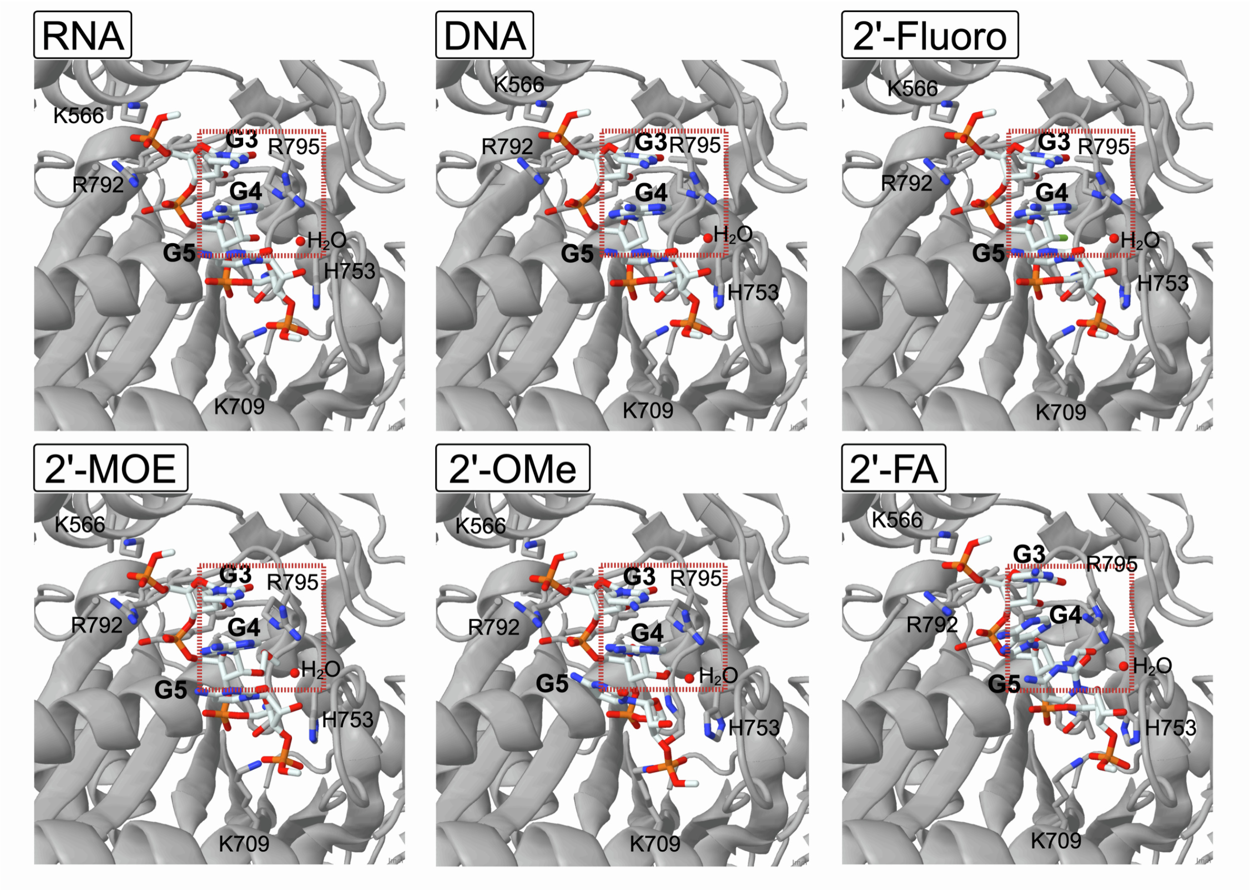


**Figure S7. Computational simulations of nucleotides at position 4.**

As the original RNA structure, Figure S4C, including the nucleotides 3, 4, and 5, was used, and DNA, 2'-Fluoro, 2'-MOE, 2'-OMe, or 2'-FA was introduced at position 4. The amino acid residues of the AGO protein used in these calculations were K566, K709, H753, Y790, R792, R795, and S798 (Table S1). The carbon atoms on AGO protein were shown in gray, while the carbon atoms of the guide RNA were shown in white. Oxygen atoms, red. Phosphorus atoms, orange. Nitrogen atoms, blue. Regions expected to be affected by chemical modifications were highlighted with red dashed squares. Enlarged structures are shown in Figure 4C.

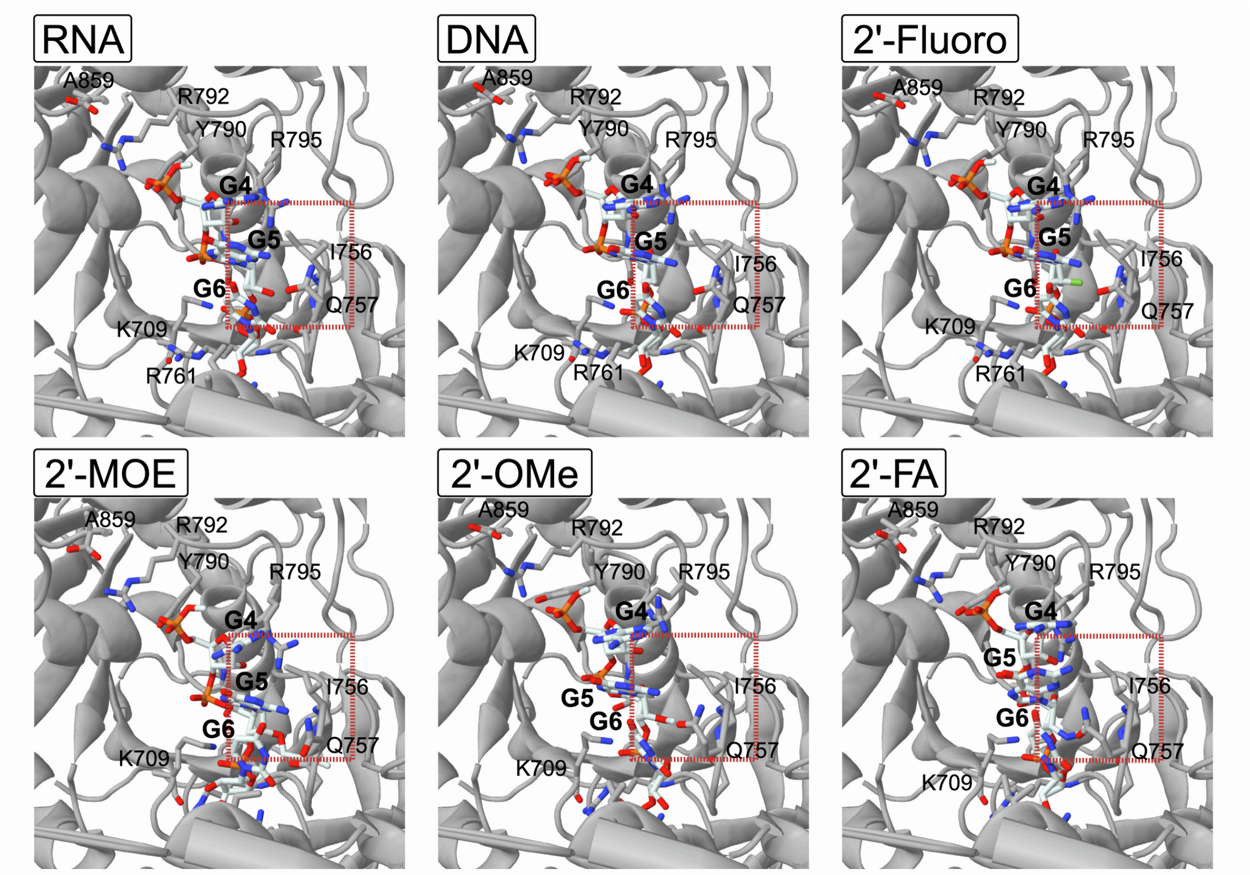


**Figure S8. Computational simulations of nucleotides at position 5.**

As the original RNA structure, Figure S4D, including the nucleotides 4, 5, and 6, was used, and DNA, 2'-Fluoro, 2'-MOE, 2'-OMe, or 2'-FA was introduced at position 5. The amino acid residues of the AGO protein used in these calculations were I365, K709, R714, G755, I756, Q757, G758, S760, R761, Y790, R792, R795, V797, S798, and A859 (Table S1). The carbon atoms on AGO protein were shown in gray, while the carbon atoms of the guide RNA were shown in white. Oxygen atoms, red. Phosphorus atoms, orange. Nitrogen atoms, blue. Regions expected to be affected by chemical modifications were highlighted with red dashed squares. Enlarged structures are shown in Figure 4D.

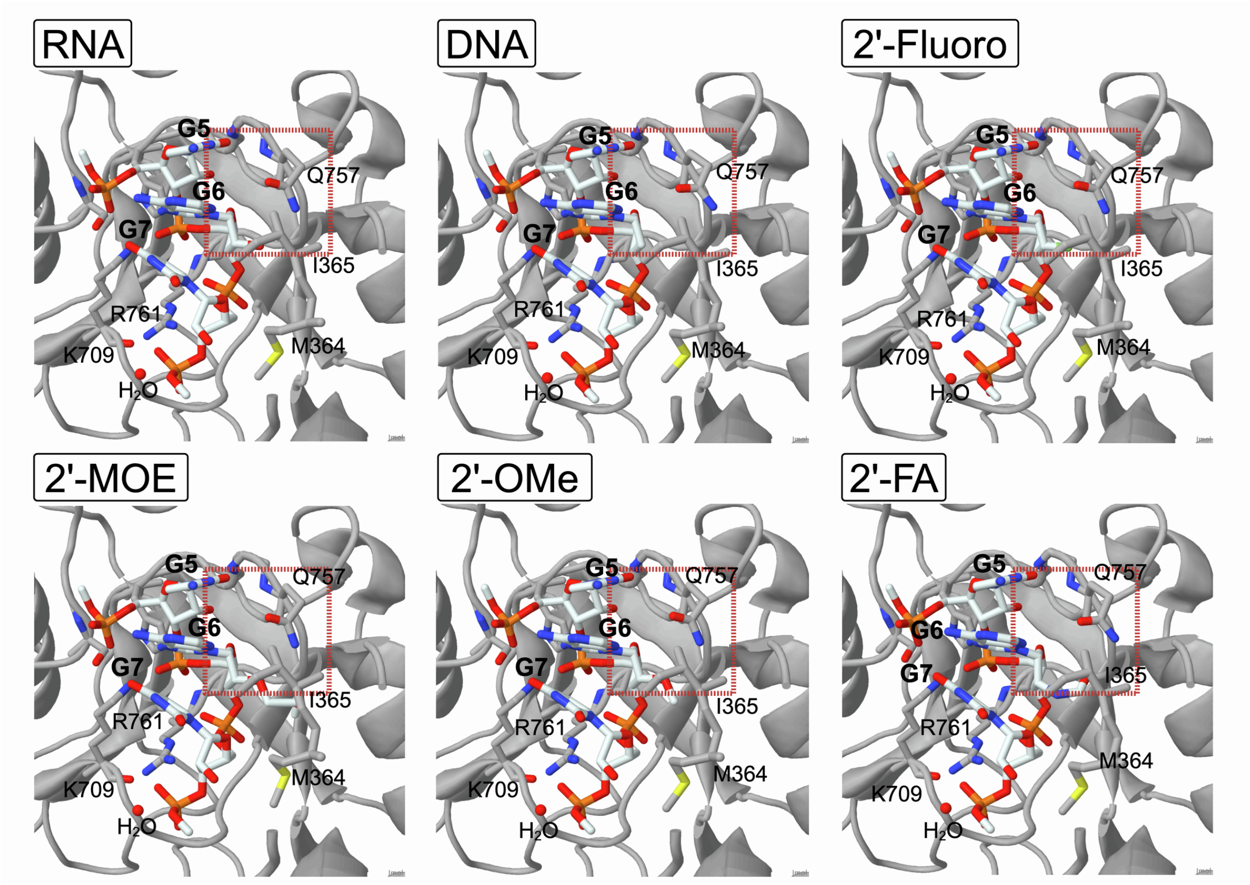


**Figure S9. Computational simulations of nucleotides at position 6.**

As the original RNA structure, Figure S4E, including the nucleotides 5, 6, and 7, was used, and DNA, 2'-Fluoro, 2'-MOE, 2'-OMe, or 2'-FA was introduced at position 6. The amino acid residues of the AGO protein used in these calculations were M364, I365, K709, H753, G755, I756, Q757, S760, R761, V797, and S798 (Table S1). The carbon atoms on AGO protein were shown in gray, while the carbon atoms of the guide RNA were shown in white. Oxygen atoms, red. Phosphorus atoms, orange. Nitrogen atoms, blue. Regions expected to be affected by chemical modifications were highlighted with red dashed squares. Enlarged structures are shown in Figure 4E.

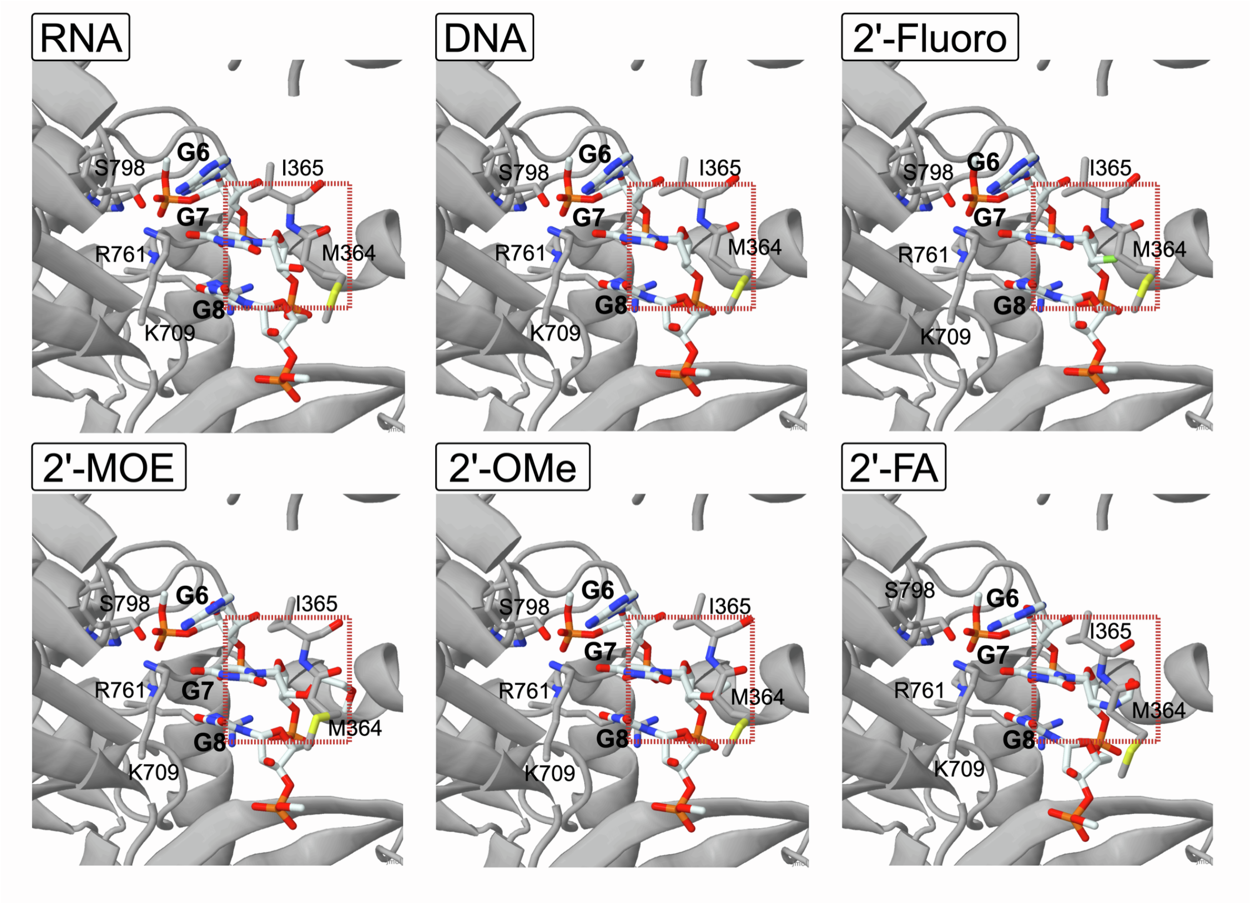


**Figure S10. Computational simulations of nucleotides at position 7.**

As the original RNA structure, Figure S4F, including the nucleotides 6, 7, and 8, was used, and DNA, 2'-Fluoro, 2'-MOE, 2'-OMe, or 2'-FA was introduced at position 7. The amino acid residues of the AGO protein used in these calculations were M364, I365, K709, H753, S760, R761, and S798 (Table S1). The carbon atoms on AGO protein were shown in gray, while the carbon atoms of the guide RNA were shown in white. Oxygen atoms, red. Phosphorus atoms, orange. Nitrogen atoms, blue. Regions expected to be affected by chemical modifications were highlighted with red dashed squares. Enlarged structures are shown in Figure 4F.

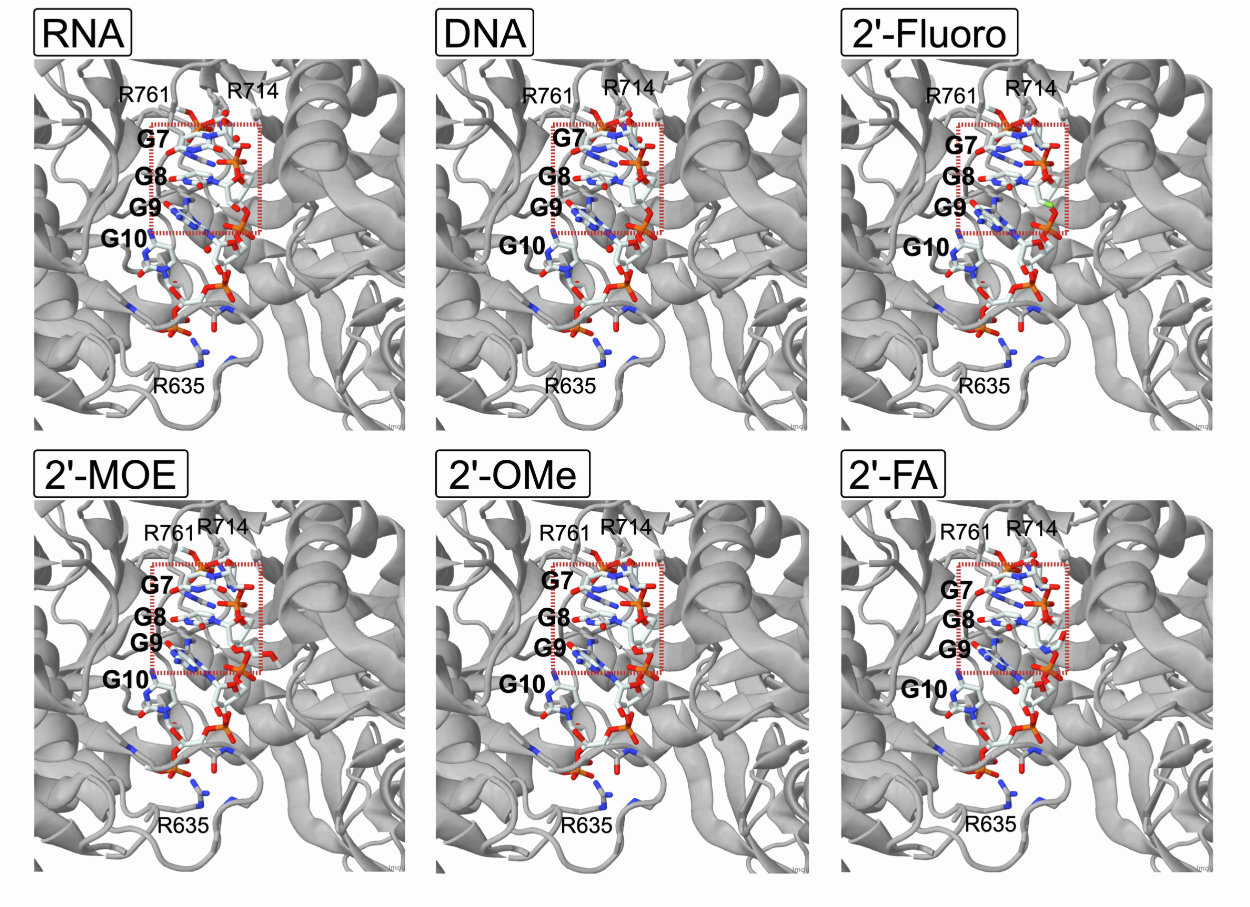


**Figure S11. Computational simulations of nucleotides at position 8.**

As the original RNA structure, Figure S4G, including the nucleotides at positions 7, 8, 9, and 10, was used, and DNA, 2'-Fluoro, 2'-MOE, 2'-OMe, or 2'-FA was introduced at position 8. The amino acid residues of the AGO protein used in these calculations were T599, H600, P601, P602, A603, G604, D605, G606, R635, R710, R714, and R761 (Table S1). The carbon atoms on AGO protein were shown in gray, while the carbon atoms of the guide RNA were shown in white. Oxygen atoms, red. Phosphorus atoms, orange. Nitrogen atoms, blue. Regions expected to be affected by chemical modifications were highlighted with red dashed squares. Enlarged structures are shown in Figure 4G.

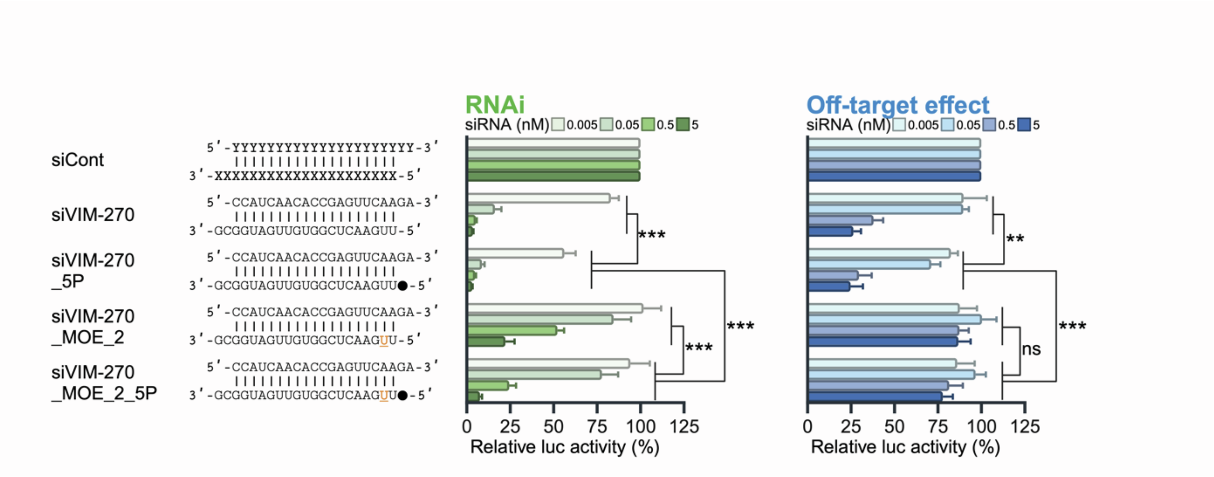


**Figure S12. Dose-dependent RNAi and off-target activities of unmodified and 2'-MOE-modified siVIM-270s with or without 5'-phosphate.**

The upper RNA strand represents the passenger strand, and the lower represents the guide strand. Green graphs depict RNAi activities, while blue graphs indicate off-target activities. In the siRNA sequence, the orange-colored characters indicate the positions of nucleotides with 2'-MOE modifications. A black circle at the 5' end represents a 5'-phosphate. RNAi/off-target activity of siRNA targeting an unrelated gene served as the control siRNA (siCont), and its value was set as 100%. *p*-values were calculated using a two-way ANOVA. Significant levels were indicated as follows: *$p<0.05$, **$p<0.01$, ***$p<0.001$. Each experiment was conducted in triplicate.

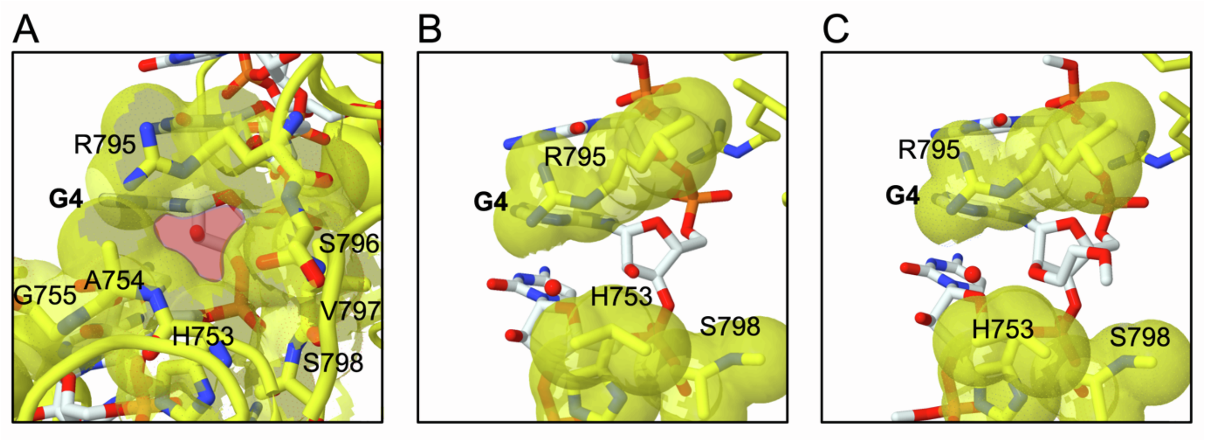


**Figure S13. Visualization of cavity-like structure formed by AGO protein.**

(**A**) A cavity-like structure (PDB ID: 4W5O) formed by amino acids of AGO protein, H753, A754, G755, R795, S796, V797, and S798. The carbon atoms of the AGO protein are shown in yellow. The van der Waals surfaces of H753, A754, G755, R795, S796, V797, and S798 are also represented by yellow, with the cavity within this region highlighted in red. (**B**), (**C**) A cavity-like structure created by H753, R795, and S798 in the simulated structure of unmodified (**B**), or in the structure with 2'-MOE at nucleotide 4 (**C**). The carbon atoms of the AGO protein are shown in yellow, and the van der Waals surfaces of H753, R795, and S798 are also represented by yellow.

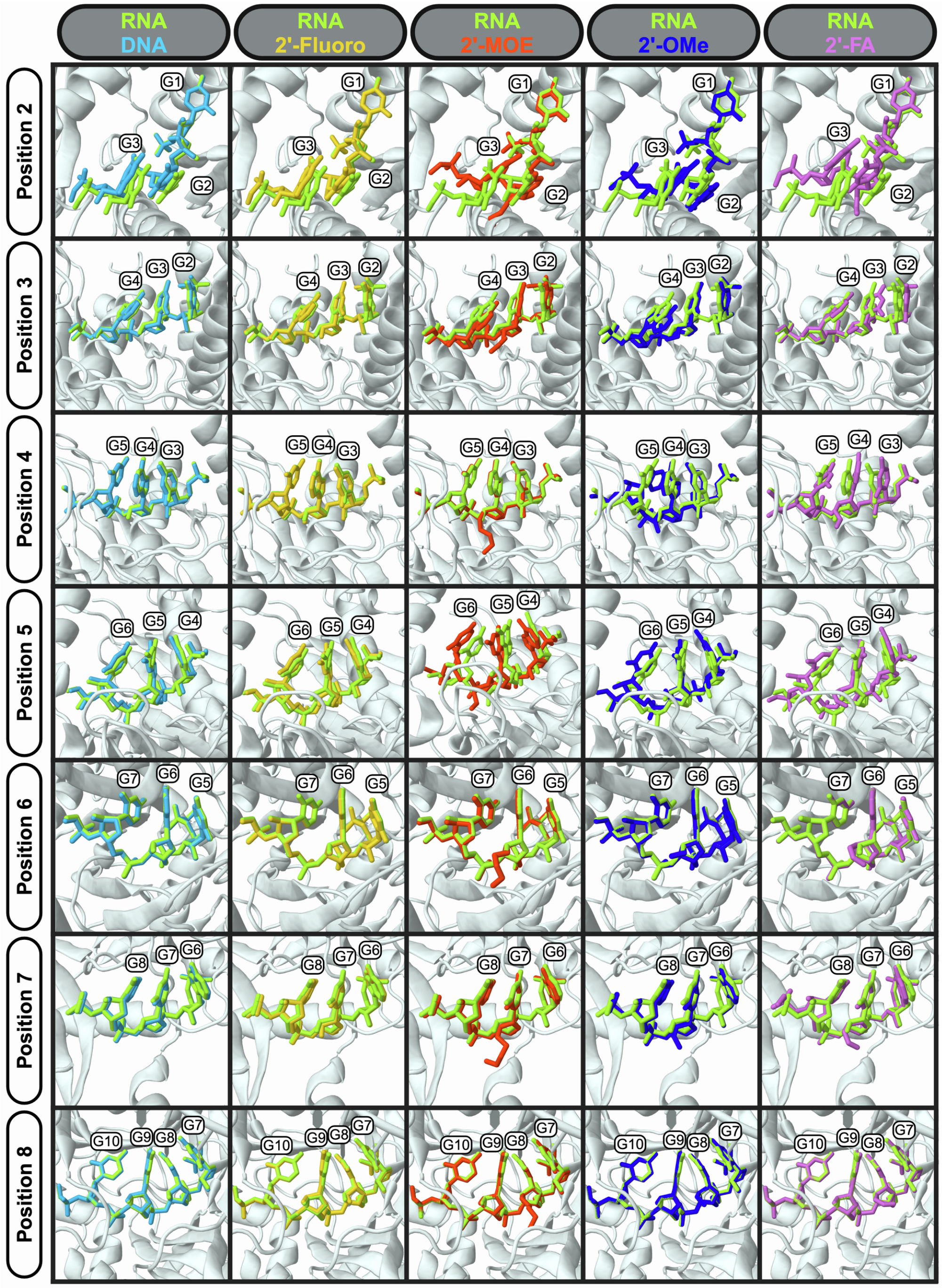


**Figure S14. The superimposed images of optimized unmodified siRNA structure and each of the simulated structures of siRNAs with chemical modifications.**

Unmodified RNA structures were shown in green, DNA in light blue, 2′-Fluoro in yellow, 2′-MOE in orange, 2′-OMe in blue, and 2′-FA in purple. Each number indicated the position of the nucleotide from the 5’ end of the guide RNA. From left to right, the structure of RNA modified with DNA, 2′-Fluoro, 2′-MOE, 2′-OMe, or 2′-FA was shown superimposed with the unmodified RNA structure. The modified position 28 was indicated from top to bottom.

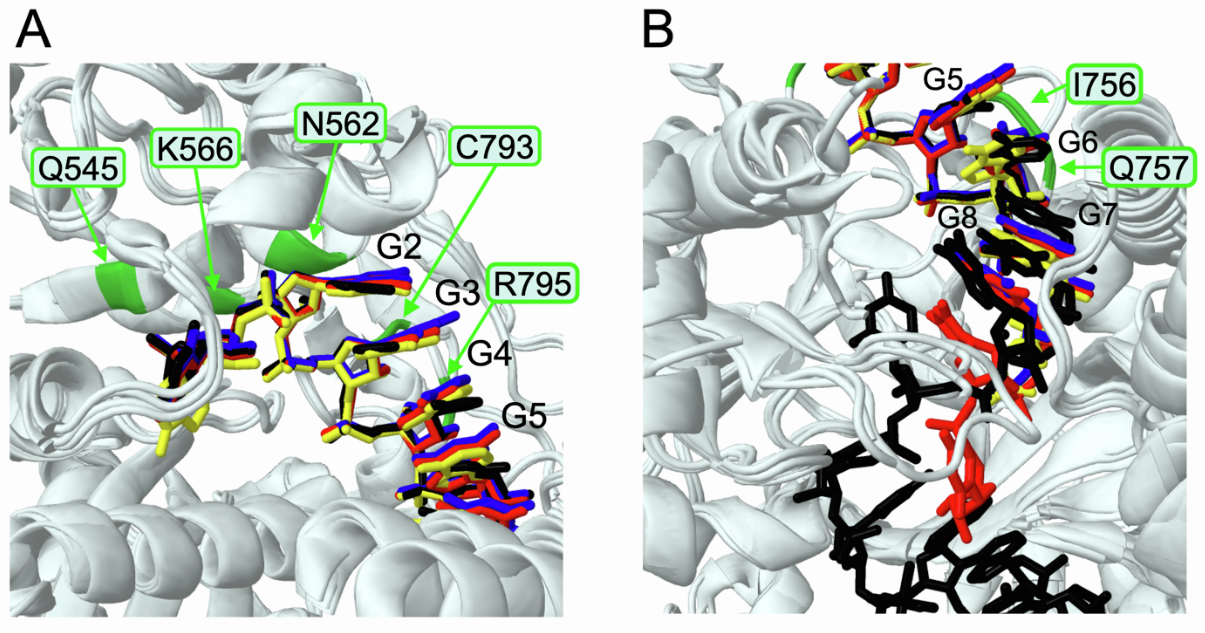


**Figure S15. Superimposed images of AGO-guide RNA complexes registered in PDB.**

The PDB structures used were as follows with nucleotide sequences in parentheses: black (PDB ID: 4W5O; 5'-UUCACAUUGCCCAAGUCUUU-3'), red (PDB ID: 4F3T; 5'-UAAAGUGCUUCAGG-3'), blue (PDB ID: 8D71; 5'-UGGAGUGUGUU-3'), and yellow (PDB ID: 4OLA; 5'-AAAAAAA-3'). (**A**) Superimposed image of AGO-guide RNA complexes at positions 2–5 of the guide RNA. The AGO protein is shown in white. The Cα atoms of the Q545, N562, K566, C793, and R795, which affect structural changes, were fixed. (**B**) Superimposed image of AGO-guide RNA complexes at positions 5–8. The Cα atoms of the I756 and Q757, which affect structural changes, were fixed.

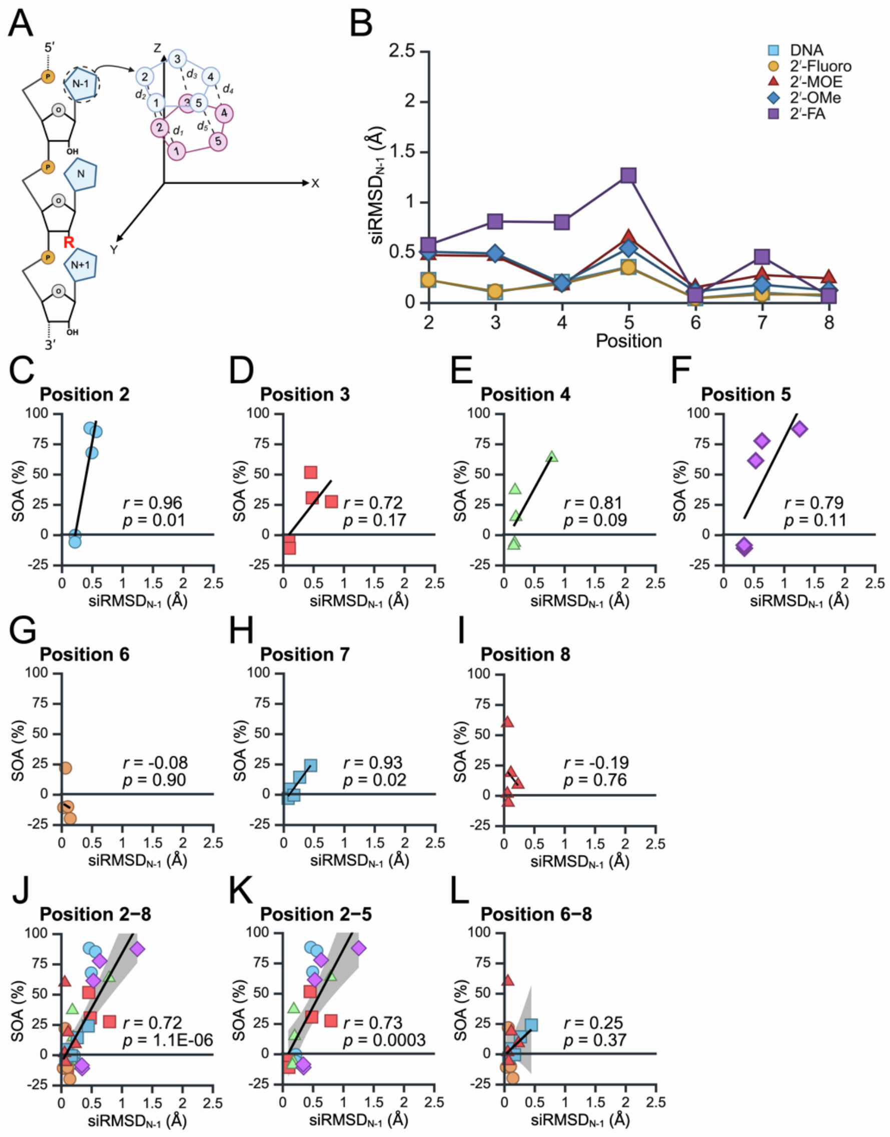


**Figure S16. Calculation of siRMSD$_{N-1}$ values and their correlations with SOAs.**

(**A**) The siRMSD$_{N-1}$value was calculated for nucleotide at position N-1, relative position to the nucleotide N, in which the 2'-ribose modification was introduced as shown as "R". (**B**) The siRMSD$_{N-1}$ value at each position with the following chemical modification: DNA (light blue), 2'-Fluoro (yellow), 2'-MOE (orange), 2'-OMe (blue), and 2'-FA (purple). The correlation between siRMSD$_{N-1}$ and SOA was shown by dot plot at each position through 2 to 8 (**C**–**I**), and positions 2–8 (**J**), 2–5 (**K**), and 6–8 (**L**). The gray-shaded areas represent the 95% confidence intervals.

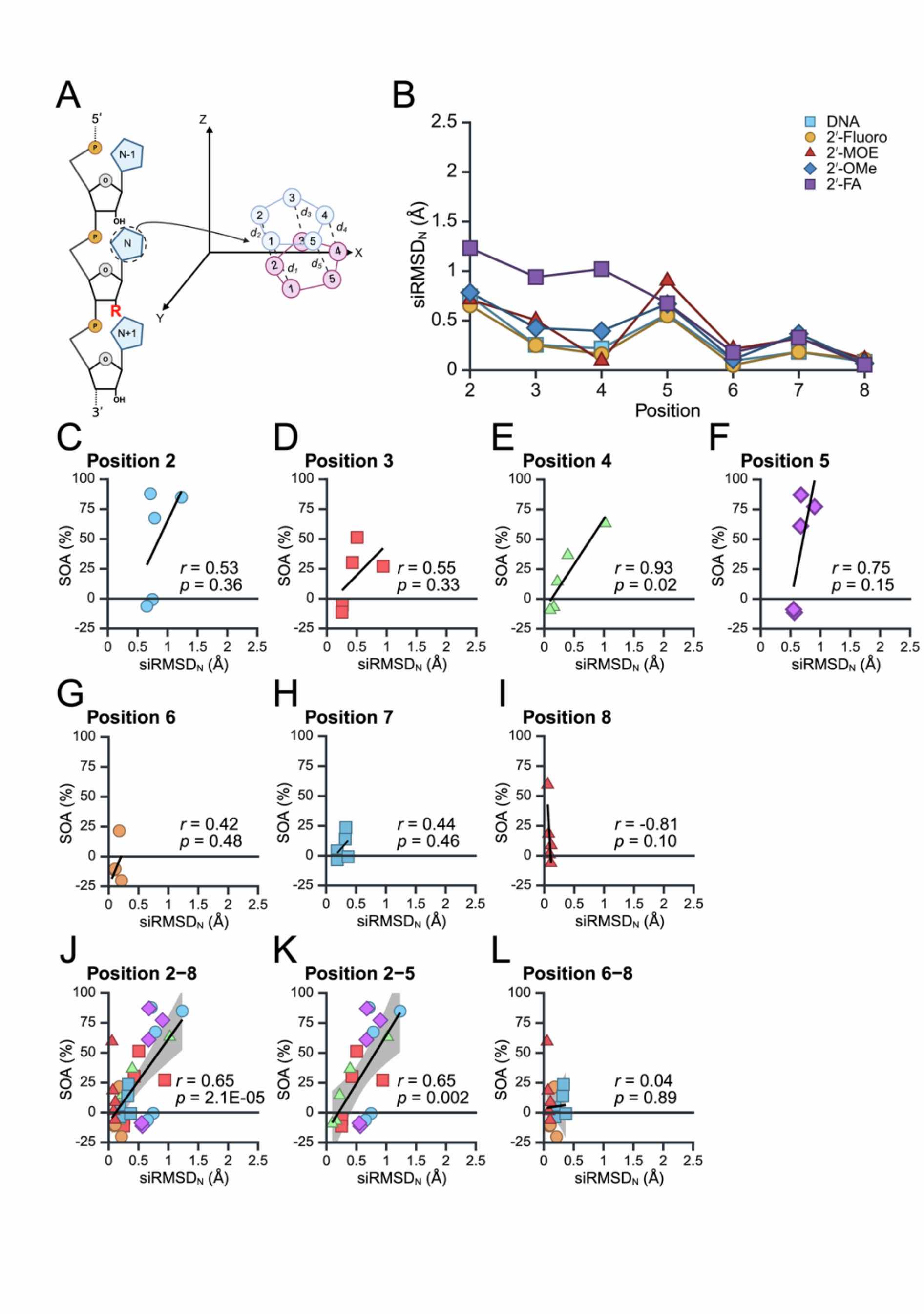


**Figure S17. Calculation of siRMSD$_N$ values and their correlations with SOAs.**

(**A**) The siRMSD$_N$ value was calculated for nucleotide at position N, in which the 2'-ribose modification was introduced as shown as "R". (**B**) The siRMSD$_N$ value at each position with the following chemical modification: DNA (light blue), 2'-Fluoro (yellow), 2'-MOE (orange), 2'-OMe (blue), and 2'-FA (purple). The correlation between siRMSD$_N$ and SOA was shown by dot plot at each position through 2 to 8 (**C**–**I**), and positions 2–8 (**J**), 2–5 (**K**), and 6–8 (**L**). The gray-shaded areas represent the 95% confidence intervals.

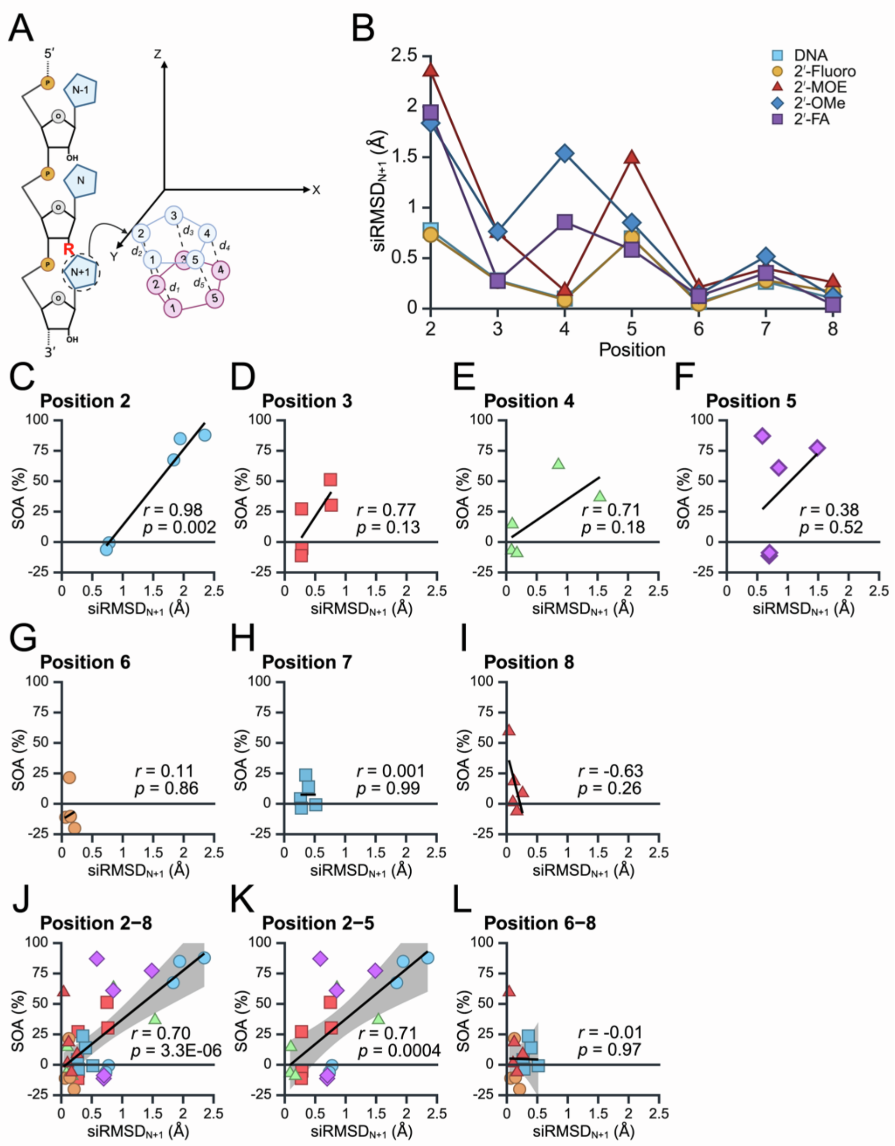


**Figure S18. Calculation of $siRMSD_{N+1}$ values and their correlations with SOAs.**

(**A**) The $siRMSD_{N+1}$value was calculated for nucleotide at position N+1. The 2'-ribose modification was introduced into the nucleotide at position N as shown as "R". (**B**) The $siRMSD_{N+1}$ value at each position with the following chemical modification: DNA (light blue), 2'-Fluoro (yellow), 2'-MOE (orange), 2'-OMe (blue), and 2'-FA (purple). The correlation between $siRMSD_{N+1}$ and SOA was shown by dot plot at each position through 2 to 8 (**C**–**I**), and positions 2–8 (**J**), 2–5 (**K**), and 6–8 (**L**). The gray-shaded areas represent the 95% confidence intervals.

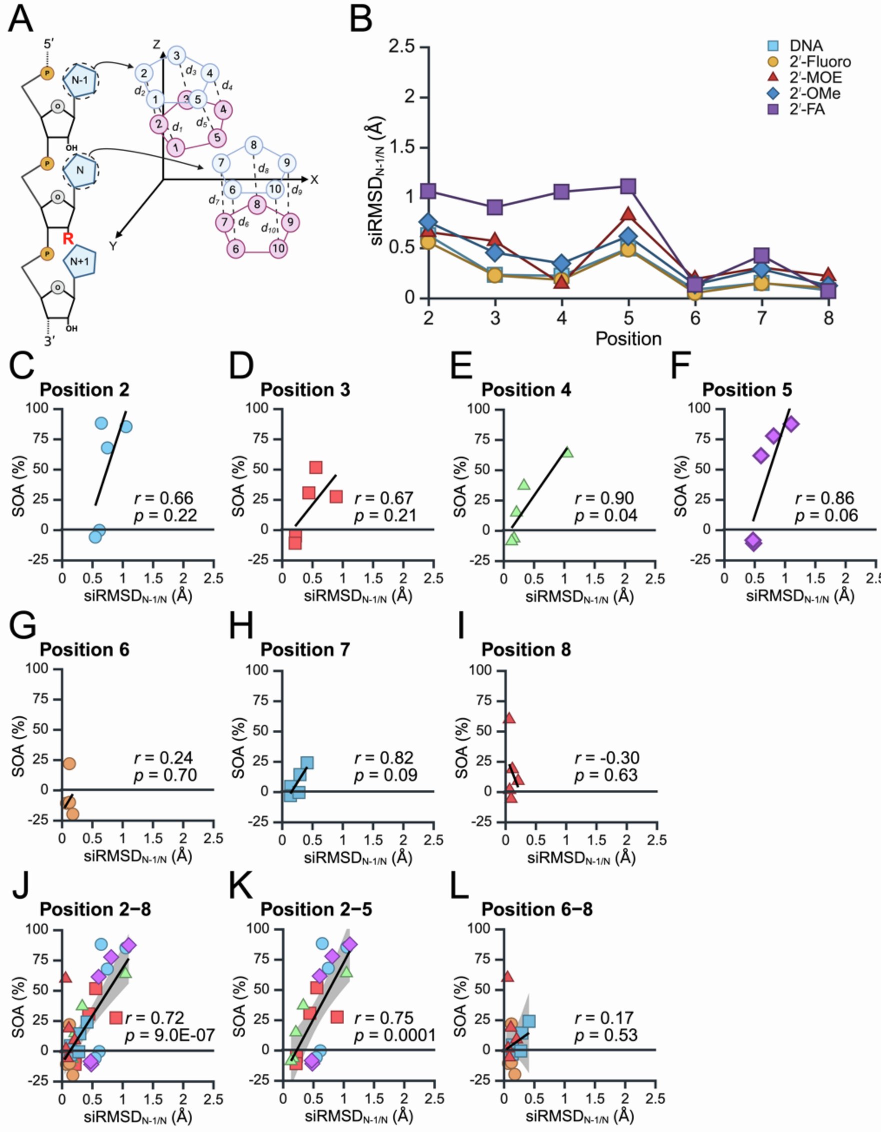

**Figure S19. Calculation of siRMSD$_{N-1/N}$ values and their correlations with SOAs.**

(**A**) The siRMSD$_{N-1/N}$ value was calculated for nucleotides at positions N-1/N. The 2'-ribose modification was introduced into the nucleotide at position N as shown as "R". (**B**) The siRMSD$_{N-1/N}$ value at each position with the following chemical modification: DNA (light blue), 2'-Fluoro (yellow), 2'-MOE (orange), 2'-OMe (blue), and 2'-FA (purple). The correlation between siRMSD$_{N-1/N}$ and SOA was shown by dot plot at each position through 2 to 8 (**C**–**I**), and positions 2–8 (**J**), 2–5 (**K**), and 6–8 (**L**). The gray-shaded areas represent the 95% confidence intervals.

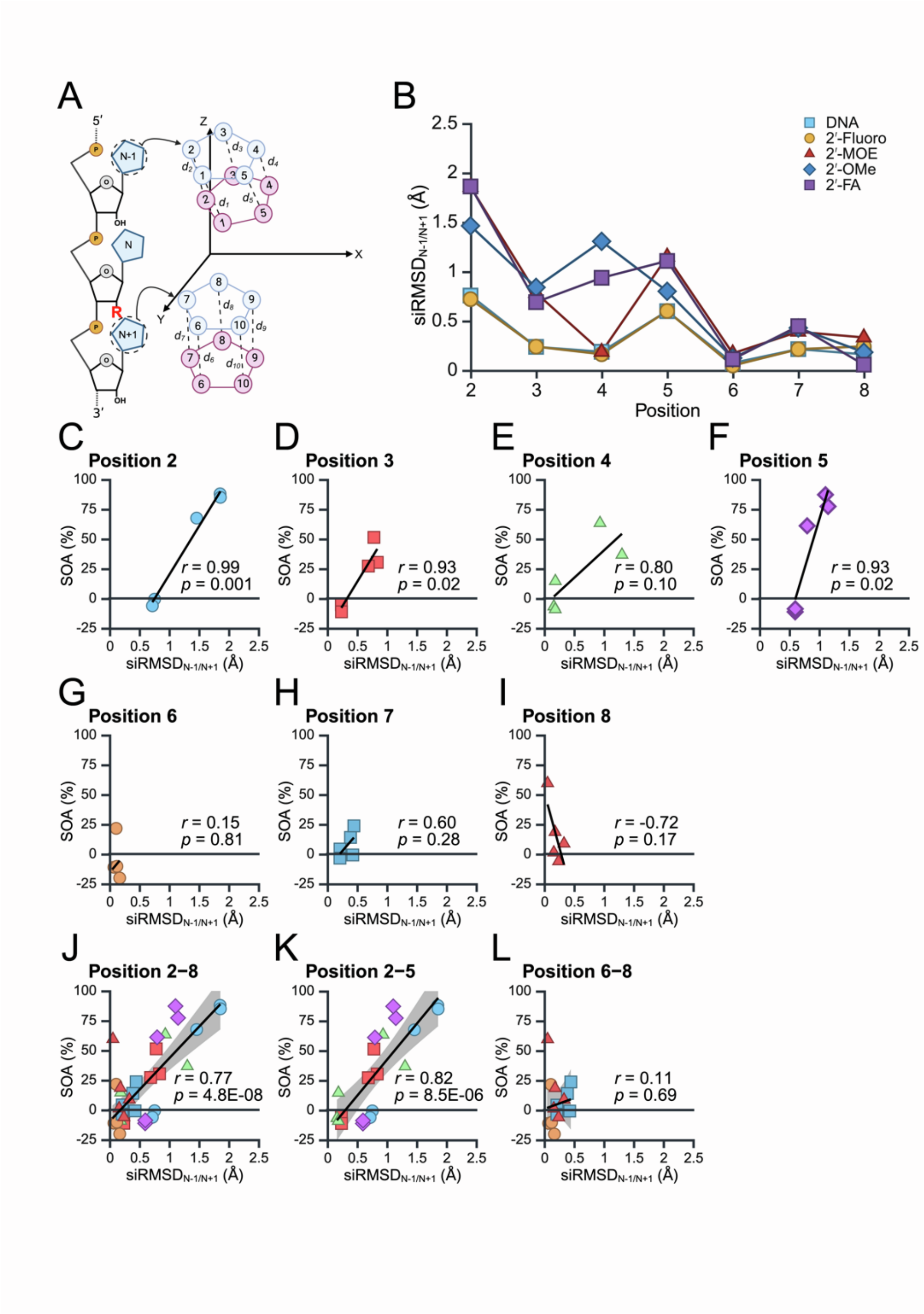


**Figure S20. Calculation of siRMSD$_{N-1/N+1}$ values and their correlations with SOAs.**

(**A**) The siRMSD$_{N-1/N+1}$ value was calculated for nucleotides at positions N-1/N+1. The 2'-ribose modification was introduced into the nucleotide at position N as shown as "R". (**B**) The siRMSD$_{N-1/N+1}$ value at each position with the following chemical modification: DNA (light blue), 2'-Fluoro (yellow), 2'-MOE (orange), 2'-OMe (blue), and 2'-FA (purple). The correlation between siRMSD$_{N-1/N+1}$ and SOA was shown by dot plot at each position through 2 to 8 (**C**–**I**), and positions 2–8 (**J**), 2–5 (**K**), and 6–8 (**L**). The gray-shaded areas represent the 95% confidence intervals.

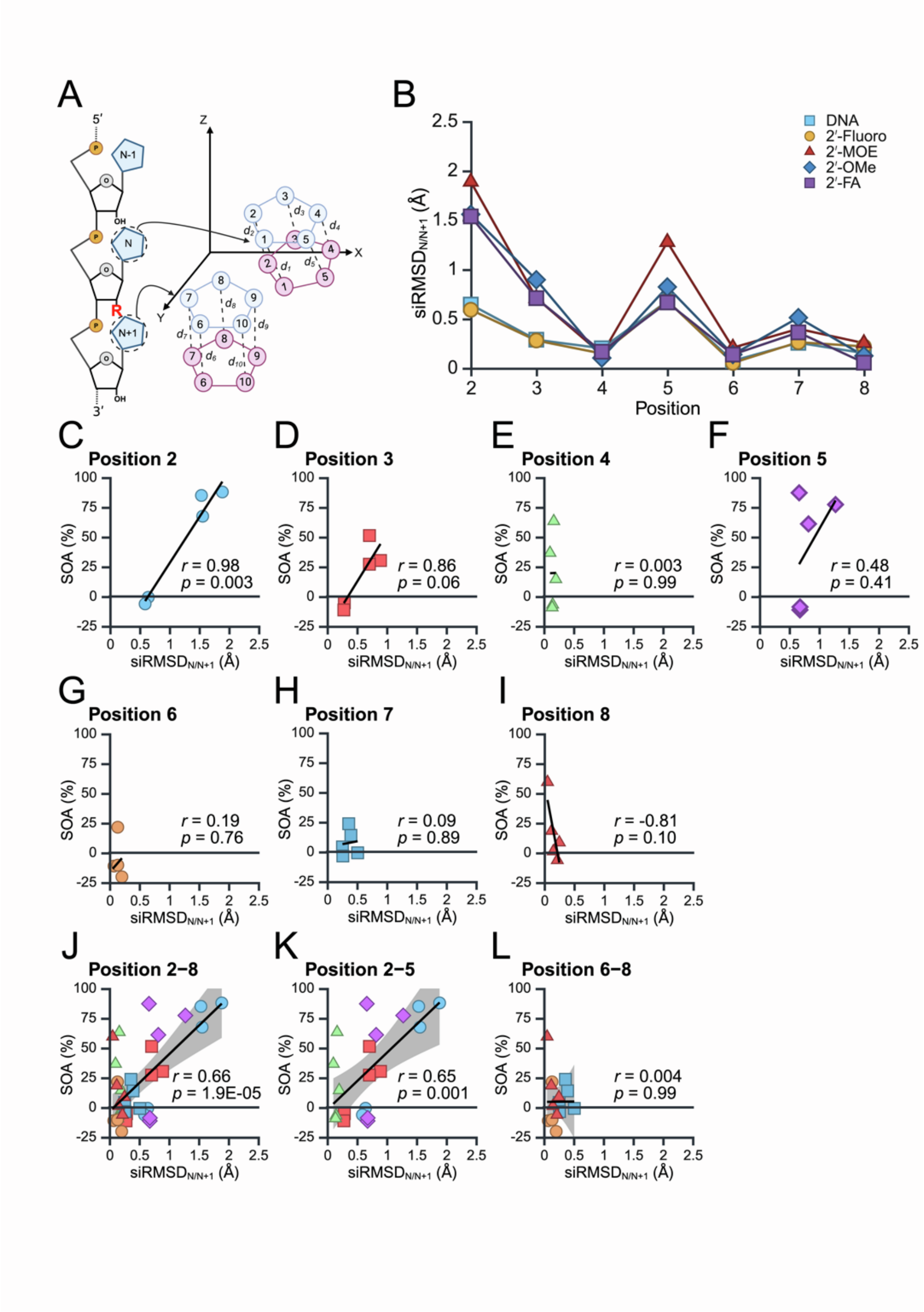


**Figure S21. Calculation of siRMSD$_{N/N+1}$ values and their correlations with SOAs.**

(**A**) The siRMSD$_{N/N+1}$ value was calculated for nucleotides at positions N/N+1. The 2'-ribose modification was introduced into the nucleotide at position N as shown as "R". (**B**) The siRMSD$_{N/N+1}$ value at each position with the following chemical modification: DNA (light blue), 2'-Fluoro (yellow), 2'-MOE (orange), 2'-OMe (blue), and 2'-FA (purple). The correlation between siRMSD$_{N/N+1}$ and SOA was shown by dot plot at each position through 2 to 8 (**C**–**I**), and positions 2–8 (**J**), 2–5 (**K**), and 6–8 (**L**). The gray-shaded areas represent the 95% confidence intervals.

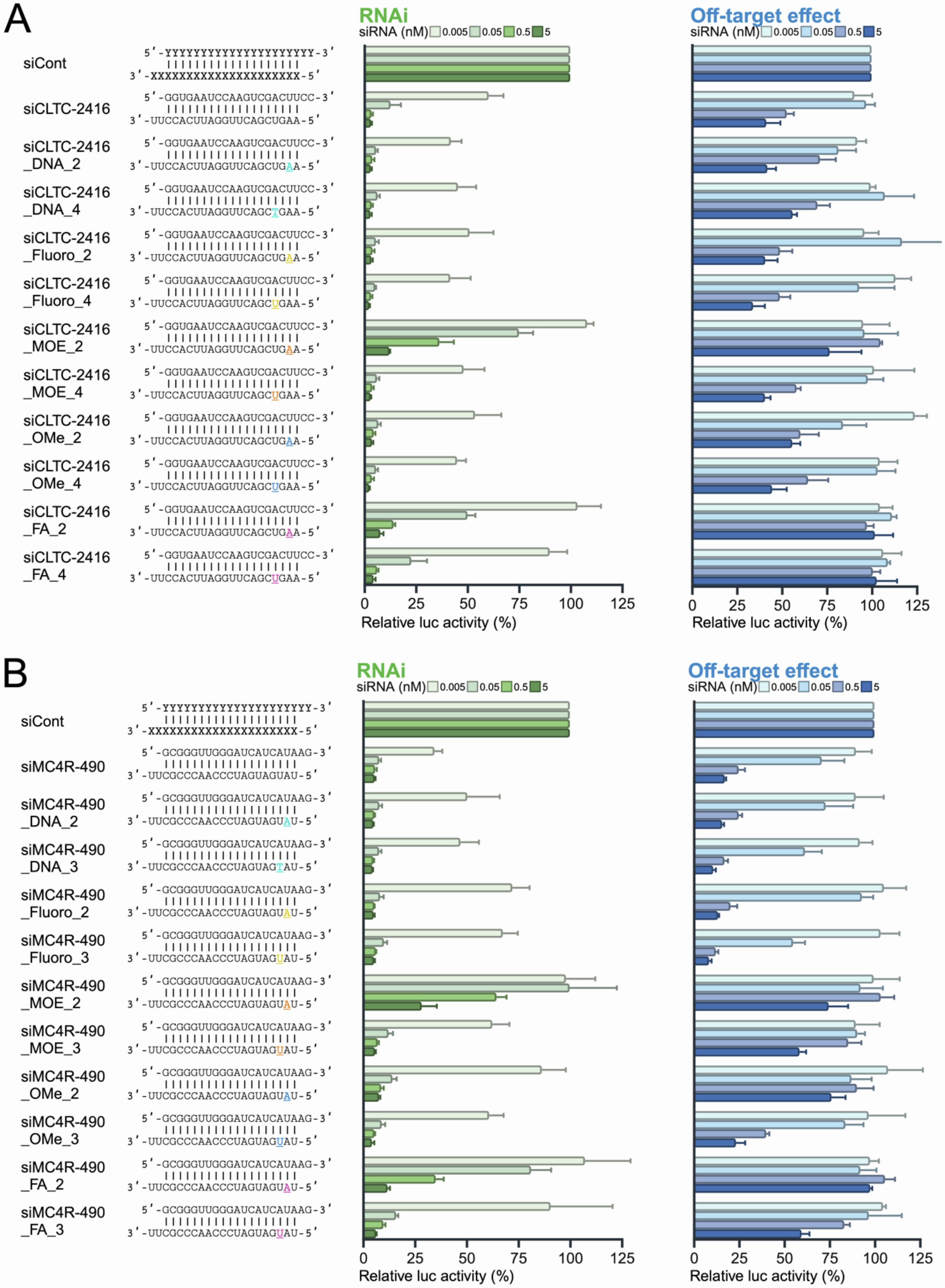
A
RNAi
siRNA (nM) 0.005 0.05 0.5 5
Off-target effect
siRNA (nM) 0.005 0.05 0.5 5
siCont
siCLTC-2416
5'-GGUGAAUCCAAGUCGACUUCC-3'
3'-UUCCACUUAGGUUCAGCUGAA-5'
siCLTC-2416 _DNA_2
3'-UUCCACUUAGGUUCAGCUGAA-5'
siCLTC-2416 _DNA_4
3'-UUCCACUUAGGUUCAGCTGAA-5'
siCLTC-2416 _Fluoro_2
siCLTC-2416 _Fluoro_4
siCLTC-2416 _MOE_2
siCLTC-2416 _MOE_4
siCLTC-2416 _OMe_2
siCLTC-2416 _OMe_4
siCLTC-2416 _FA_2
siCLTC-2416 _FA_4
0 25 50 75 100 125
Relative luc activity (%)
B
RNAi
Off-target effect
siCont
siMC4R-490
5'-GCGGGUUGGGAUCAUCAUAAG-3'
3'-UUCGCCCAACCCUAGUAGUAU-5'
siMC4R-490 _DNA_2
siMC4R-490 _DNA_3
3'-UUCGCCCAACCCUAGUAGTAU-5'
siMC4R-490 _Fluoro_2
siMC4R-490 _Fluoro_3
siMC4R-490 _MOE_2
siMC4R-490 _MOE_3
siMC4R-490 _OMe_2
siMC4R-490 _OMe_3
siMC4R-490 _FA_2
siMC4R-490 _FA_3
0 25 50 75 100 125
Relative luc activity (%)

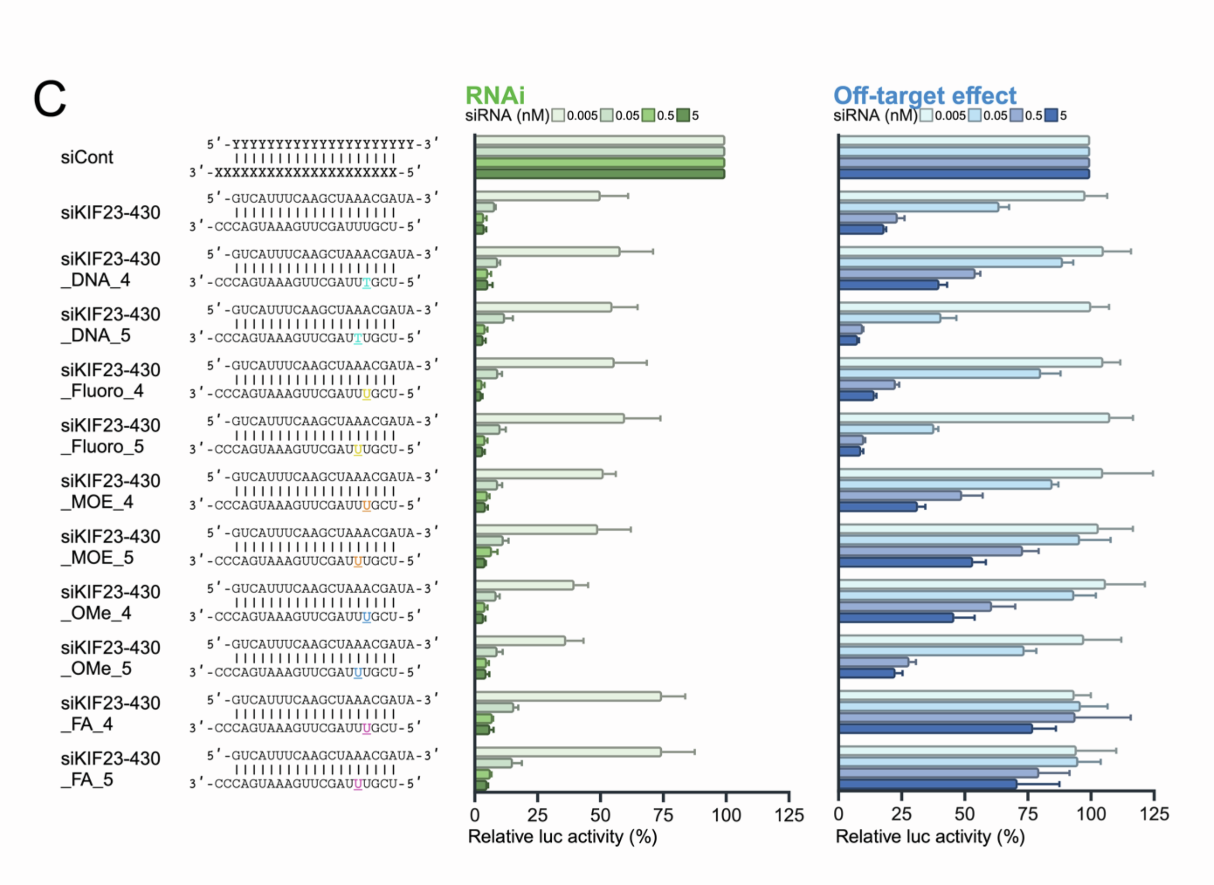


**Figure S22. Dose-dependent RNAi and off-target activities of siRNAs targeting (A) *CLTC*, (B) *MC4R*, and (C) *KIF23* with and without 2'-ribose modifications.**

The upper RNA strand represents the passenger strand, and the lower represents the guide strand. Green graphs indicate RNAi activities, while blue graphs indicate off-target activities. In the siRNA sequence, the colored characters indicate the positions of nucleotides with modifications: light blue, DNA; yellow, 2'-Fluoro; orange, 2'-MOE; blue, 2'-OMe; and purple, 2'-FA. Numbers following the names of chemical modifications denote the positions where the modifications were introduced. RNAi/off-target activity of siRNA targeting unrelated gene served as the control siRNA (siCont), and its value was set as 100%. Each experiment was conducted in triplicate.

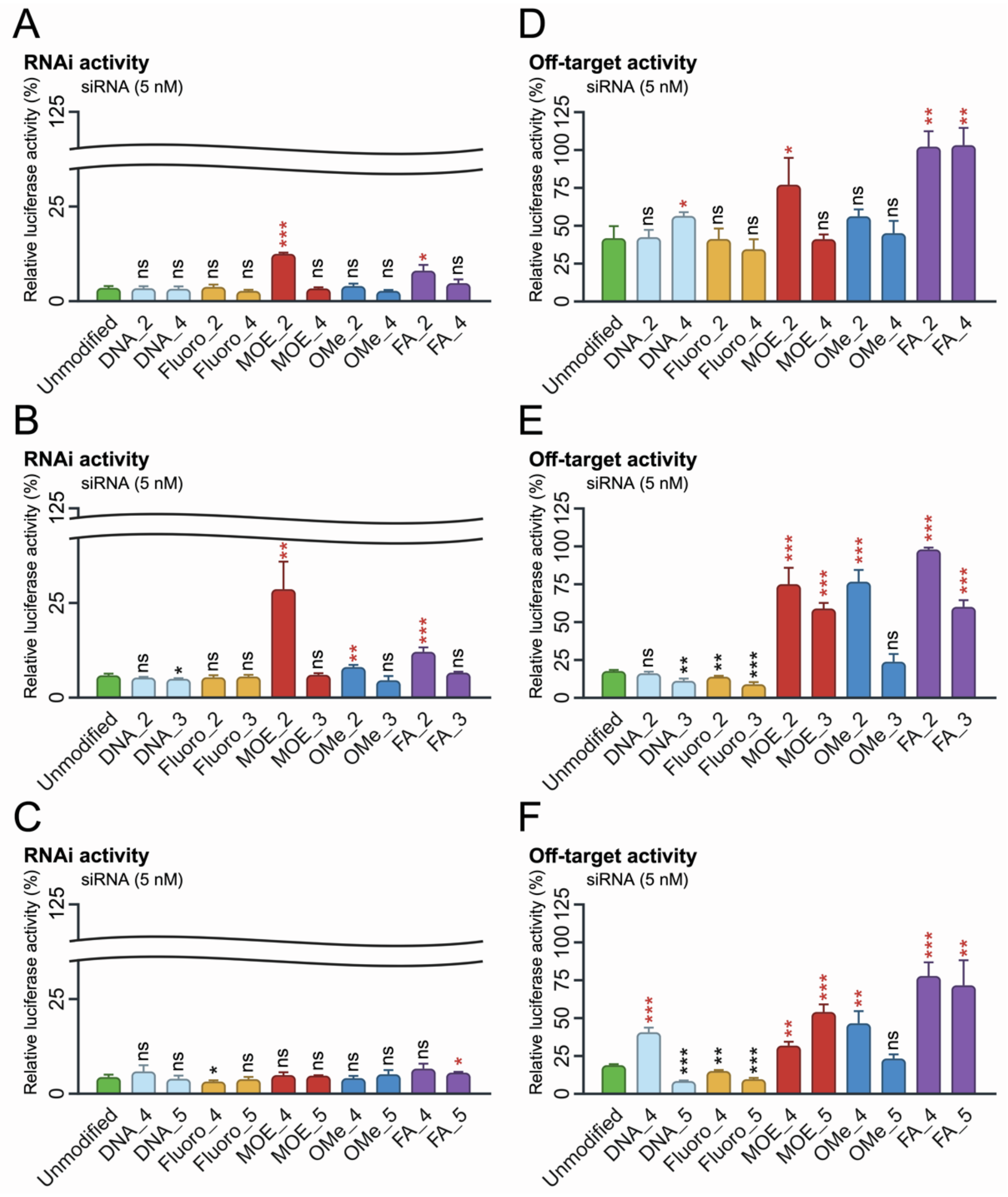


**Figure S23. RNAi and off-target activities for siRNAs targeting (A, D) *CLTC*, (B, E) *MC4R*, and (C, F) *KIF23* with and without 2' chemical modifications.**

(**A**–**C**) RNAi activities and (**D**–**F**) off-target activities of siRNAs at the concentration of 5 nM extracted from Figure S22. The vertical bars represent relative luciferase activity, normalized to siCont, while the horizontal bars indicate the type of chemical modifications and their positions. Green bar indicates the result of unmodified siRNA; light blue, DNA; yellow, 2'-Fluoro; red, 2'-MOE; dark blue, 2'-OMe: purple, 2'-FA. Modified siRNAs were compared to their unmodified counterparts using the t-test, with significance levels indicated as follows: $*p < 0.05$, $**p < 0.01$, $***p < 0.001$. A red asterisk denotes a significant decrease in activity compared to the unmodified counterpart, whereas a black asterisk indicates a significant increase in activity. Each experiment was conducted in triplicate.

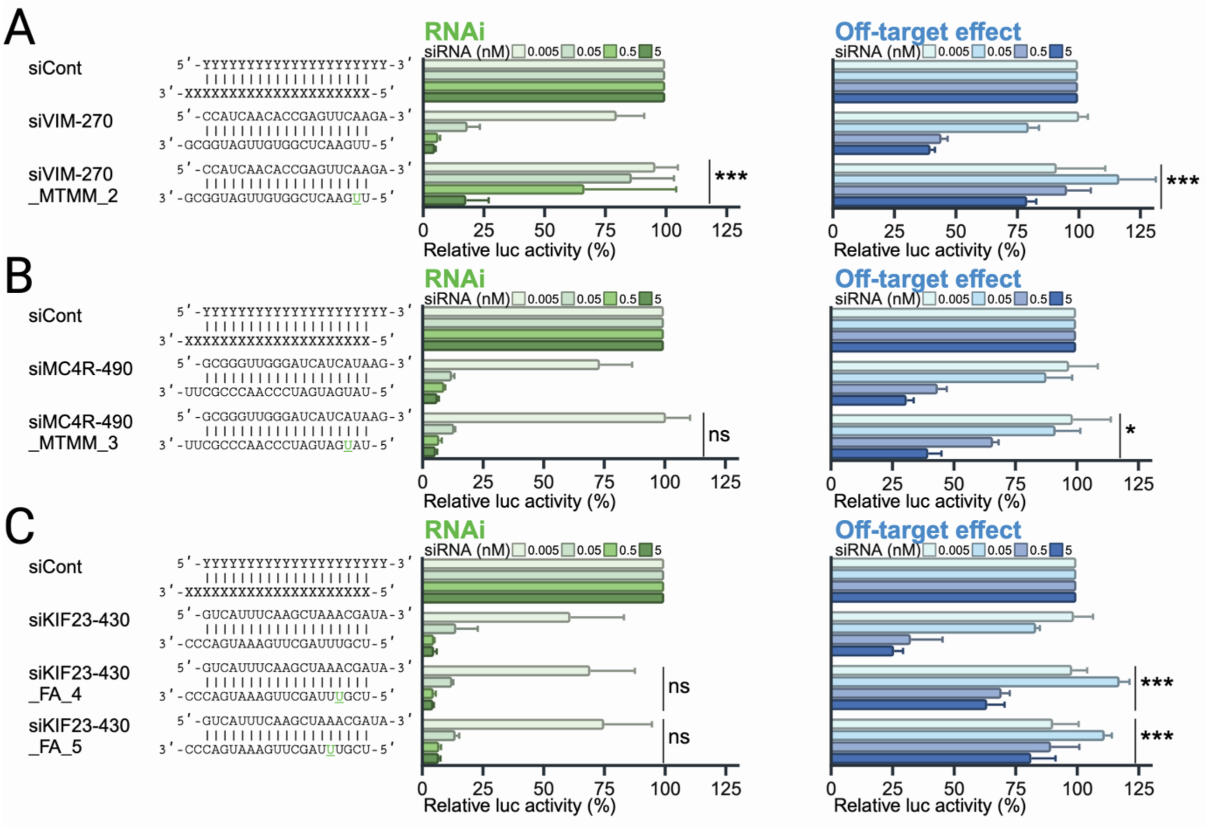


**Figure S24. Dose-dependent RNAi and off-target activities for siRNAs targeting (A) *vimentin*, (B) *MC4R*, and (C) *KIF23* with and without 2'-MTMM modifications.**

The upper RNA strand represents the passenger strand, while the lower strand represents the guide strand. Green graphs display RNAi activities, while blue graphs indicate off-target effects. In the RNA sequence, a green underline marks the position of the 2'-MTMM modification. Relative luciferase activity (%) was calculated by comparing *Renilla* luciferase activity to firefly luciferase activity. siRNA targeting unrelated gene, *green fluorescent protein* (*GFP*), was used as a control siRNA (siCont). *p*-values were determined using a two-way ANOVA, with significance levels indicated as *$p<0.05$, **$p<0.01$, and ***$p<0.001$.

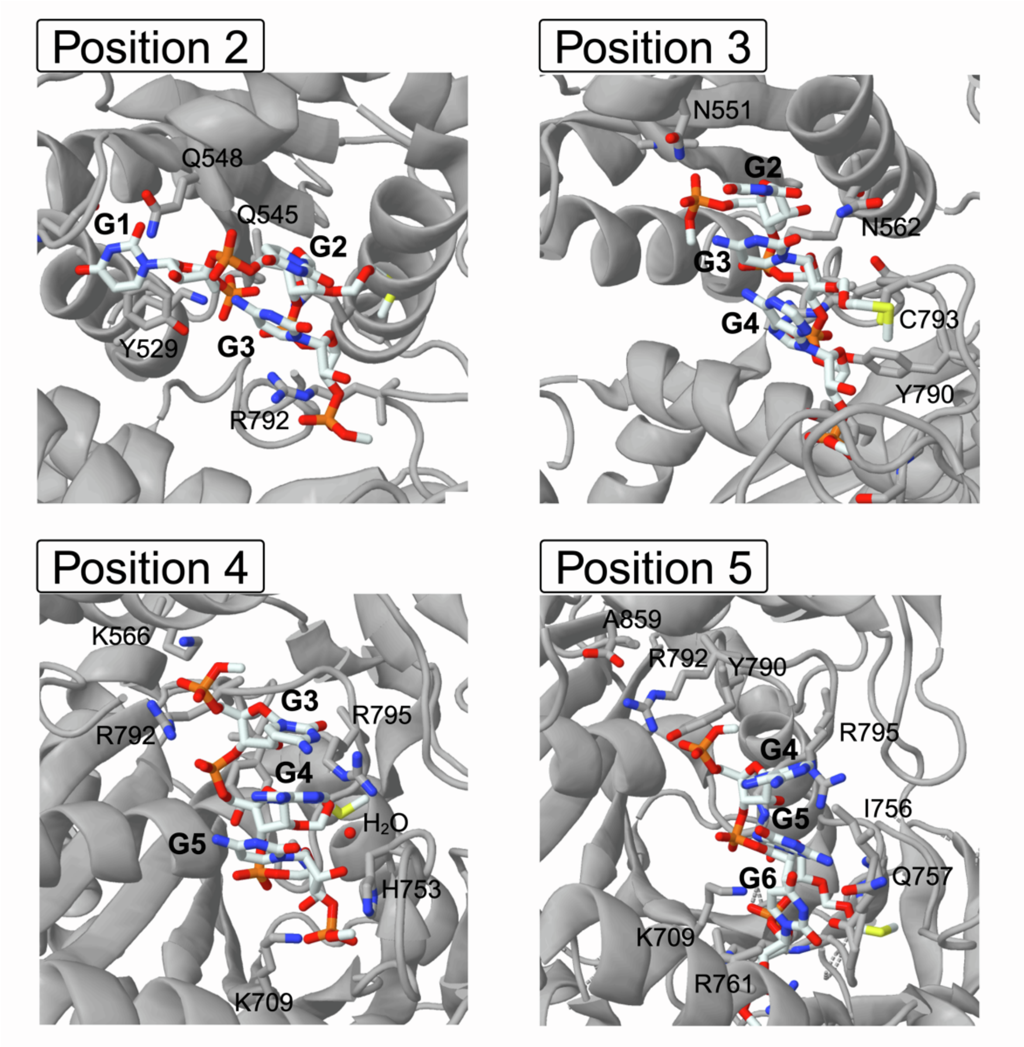


**Figure S25. The results of computational simulations of siRNA guide strands with 2'-MTMM modifications on AGO2 protein.**

The structure of siRNA with 2'-MTMM modification at each of positions 2–5, using the original RNA structure depicted in Figure S4, A to D. The carbon atoms of AGO2 protein were shown in gray, while the carbon atoms of RNA are in white. Oxygen atoms are colored red, phosphorus atoms are orange, nitrogen atoms are blue, and sulfur atoms are yellow.

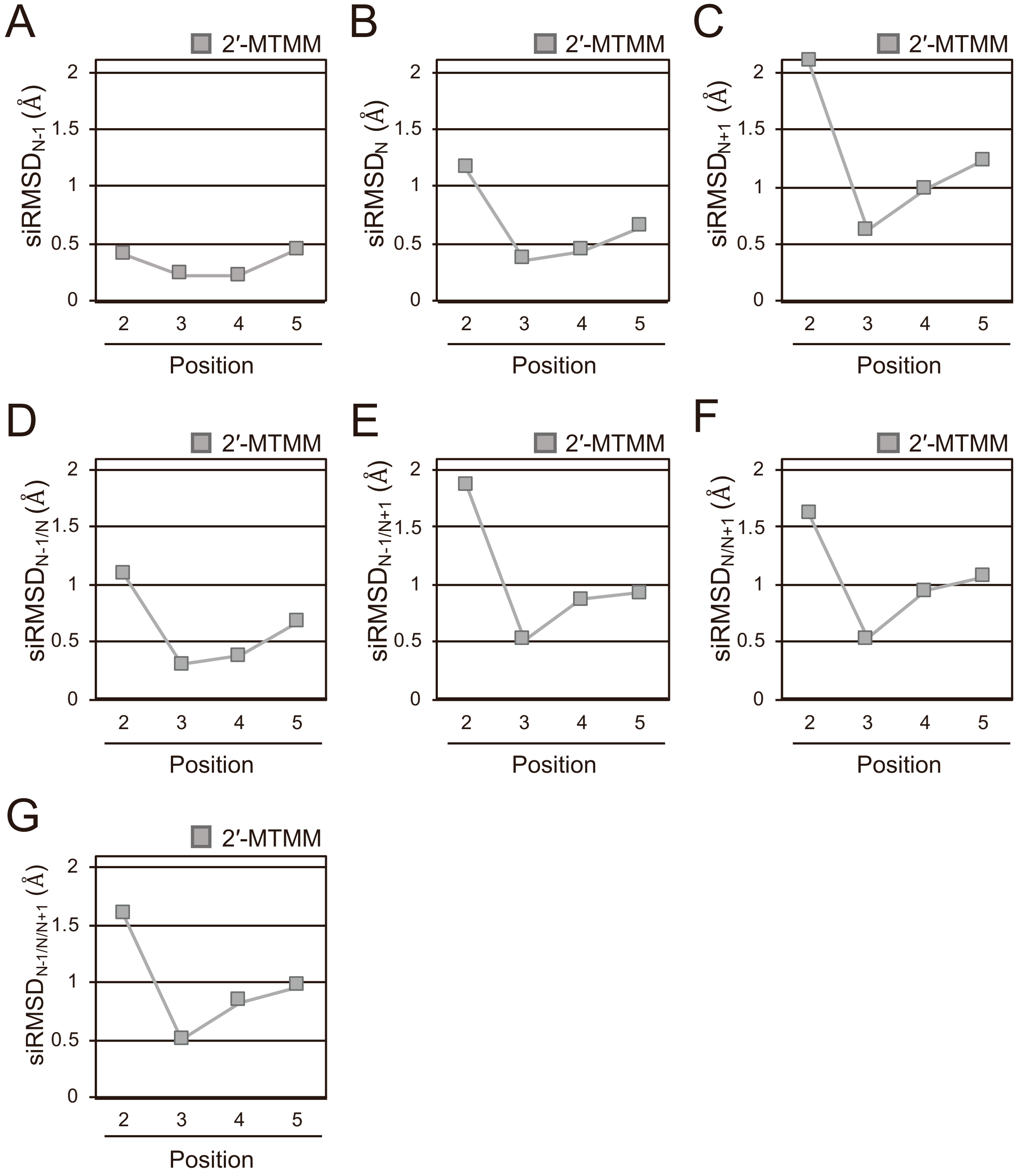


**Figure S26. Calculated siRMSD values at each position with 2'-MTMM modification.**
(**A**) siRMSD$_{N-1}$, (**B**) siRMSD$_{N}$, (**C**) siRMSD$_{N+1}$, (**D**)siRMSD$_{N-1/N}$, (**E**) siRMSD$_{N-1/N+1}$, (**F**) siRMSD$_{N/N+1}$, and (**G**) siRMSD$_{N-1/N/N+1}$. The x-axis represented the positions in the seed region where the 2'-MTMM modification was introduced, and the y-axis showed the siRMSD value.

## Table S1. Amino acids of AGO2 protein used in the calculation

| PDB ID | Nucleic Sequence | Amino Acids |
|---|---|---|
| 4W5O | 5′-U(1)U(2)C(3)-3′ | [GLY]524, [LYS]525, [THR]526, [TYR]529, [LYS]533, [GLN]545, [GLN]548, [LYS]566, [ARG]792 |
| 4W5O | 5′-U(2)C(3)A(4)-3′ | [VAL]547, [GLN]548, [ASN]551, [ASN]562, [LYS]566, [TYR]790, [ARG]792, [CYS]793, [VAL]797, [SER]798, [TYR]804, [ALA]859 |
| 4W5O | 5′-C(3)A(4)C(5)-3′ | [LYS]566, [LYS]709, [HIS]753, [TYR]790, [ARG]792, [ARG]795, [SER]798 |
| 4F3T | 5′-A(4)G(5)U(6)-3′ | [ILE]365, [LYS]709, [ARG]714, [GLY]755, [ILE]756, [GLN]757, [GLY]758, [SER]760, [ARG]761, [TYR]790, [ARG]792, [ARG]795, [VAL]797, [SER]798, [ALA]859 |
| 4W5O | 5′-C(5)A(6)U(7)-3′ | [MET]364, [ILE]365, [LYS]709, [HIS]753, [GLY]755, [ILE]756, [GLN]757, [SER]760, [ARG]761, [VAL]797, [SER]798 |
| 4W5O | 5′-A(6)U(7)U(8)-3′ | [MET]364, [ILE]365 [LYS]709, [HIS]753, [SER]760, [ARG]761, [SER]798 |
| 4W5O | 5′-U(7)U(8)G(9)C(10)-3′ | [THR]599, [HIS]600, [PRO]601, [PRO]602, [ALA]603, [GLY]604, [ASP]605, [GLY]606, [ARG]635, [ARG]710, [ARG]714, [ARG]761 |

Note: U = uridine; C = cytosine; A = adenine; G = guanine
The number following each amino acid name indicates the position of the amino acid from the N-terminus of AGO2 protein.

**Table S2. Nucleotide sequences of siRNAs**
(The full table is provided as a separate Excel file)

## Table S3. Inserted oligonucleotide sequences in psiCHECK-reporters

| Oligonucleotide name | Sequence (5′ → 3′) |
|---|---|
| siVIM-270_CM_s | tcgagCGCCATCAACACCGAGTTCAAGAg |
| siVIM-270_CM_as | aattcTCTTGAACTCGGTGTTGATGGCGc |
| siVIM-270_SM_s | tcgagGCGGTAGTTGTGGGAGTTCAAGAGCGGTAGTTGTGGGAGTTCAAGAGCGGTAGTTGTGGGAGTTCAAGAg |
| siVIM-270_SM_as | aattcTCTTGAACTCCCACAACTACCGCTCTTGAACTCCCACAACTACCGCTCTTGAACTCCCACAACTACCGCc |
| siMC4R-490_CM_s | tcgagAAGCGGGTTGGGATCATCATAAGg |
| siMC4R-490_CM_as | aattcCTTATGATGATCCCAACCCGCTTc |
| siMC4R-490_SM_s | tcgagTTCGCCCAACCCTTCATCATAAGTTCGCCCAACCCTTCATCATAAGTTCGCCCAACCCTTCATCATAAGg |
| siMC4R-490_SM_as | aattcCTTATGATGAAGGGTTGGGCGAACTTATGATGAAGGGTTGGGCGAACTTATGATGAAGGGTTGGGCGAAc |
| siCLTC-2416_CM_s | tcgagAAGGTGAATCCAAGTCGACTTCCg |
| siCLTC-2416_CM_as | aattcGGAAGTCGACTTGGATTCACCTTc |
| siCLTC-2416_SM_s | tcgagTTCCACTTAGGTTGTCGACTTCCTTCCACTTAGGTTGTCGACTTCCTTCCACTTAGGTTGTCGACTTCCg |
| siCLTC-2416_SM_as | aattcGGAAGTCGACAACCTAAGTGGAAGGAAGTCGACAACCTAAGTGGAAGGAAGTCGACAACCTAAGTGGAAc |
| siKIF23-430_CM_s | tcgagGGGTCATTTCAAGCTAAACGATAg |
| siKIF23-430_CM_as | aattcTUTCGTTTUGCTTGUUUTGUCCCc |
| siKIF23-430_SM_s | tcgagCCCAGTAAAGTTCCTAAACGATACCCAGTAAAGTTCCTAAACGATACCCAGTAAAGTTCCTAAACGATAg |
| siKIF23-430_SM_as | aattcTATCGTTTAGGAACTTTACTGGGTATCGTTTAGGAACTTTACTGGGTATCGTTTAGGAACTTTACTGGGc |

Note: _s = sense strand; _as = antisense strand
_CM = complete match; _SM = seed match
Underline = complementary sequence with seed region of the siRNA guide strand
Double underline = complementary sequence with the siRNA guide strand
Lower case = sequence of restriction enzyme site

**Table S4. Primer sequences for qRT-PCR**

| Primer name | Sequence (5′ → 3′) |
|---|---|
| GAPDH_F | TGCACCACCAACTGCTTAG |
| GAPDH_R | AGAGGCAGGGATGATGTTC |
| VIM_F | CAGGACTCGGTGGACTTCTC |
| VIM_R | GTCGATGTAGTTGGCGAAGC |
| VAPA_F | TTCAGGAAATGCCAAGAGGT |
| VAPA_R | TCAACAACTGCCTCACAAGG |
| MTPN_F | TAGGTGCAGTGTGTGGAAGC |
| MTPN_R | TGCATGGAAGAAAACAGCAG |
| PTPRF_F | CTGGTTTGCAGCTGTTTTCA |
| PTPRF_R | CCTCAGCAAGCTGGGATAAT |
| SURF4_F | GTCAAGGTTGGTTGGCTGAT |
| SURF4_R | GCCAGGAGAAACAGGAACAC |

Note: _F = forward primer; _R = reverse primer

**Table S5. Total energies of optimized structures**

(The full table is provided as a separate Excel file)

**Table S6. Cartesian coordinates of optimized structures**

All the calculation is performed at the ωB97X-D/6-31G(d) level of theory

(The full table is provided as a separate Excel file)
